# AMBWO: An Augmented Multi-Strategy Beluga Whale Optimization for Numerical Optimization Problems

**DOI:** 10.3390/biomimetics9120727

**Published:** 2024-11-28

**Authors:** Guoping You, Zengtong Lu, Zhipeng Qiu, Hao Cheng

**Affiliations:** 1 School of Information Engineering, Jiangxi Science and Technology Normal University, Nanchang 330000, China; bluesky140@163.com; 2 Ruijie Networks Co., Ltd., Fuzhou 350000, China; 3 School of Computer Science and Information Security, Guilin University of Electronic Technology, Guilin 541000, China; chenghao@guet.edu.cn; 4 College of Computer and Cyber Security, Fujian Normal University, Fuzhou 350117, China

**Keywords:** beluga whale optimization, adaptive, metaheuristic, global optimization

## Abstract

Beluga whale optimization (BWO) is a swarm-based metaheuristic algorithm inspired by the group behavior of beluga whales. BWO suffers from drawbacks such as an insufficient exploration capability and the tendency to fall into local optima. To address these shortcomings, this paper proposes augmented multi-strategy beluga optimization (AMBWO). The adaptive population learning strategy is proposed to improve the global exploration capability of BWO. The introduction of the roulette equilibrium selection strategy allows BWO to have more reference points to choose among during the exploitation phase, which enhances the flexibility of the algorithm. In addition, the adaptive avoidance strategy improves the algorithm’s ability to escape from local optima and enriches the population quality. In order to validate the performance of the proposed AMBWO, extensive evaluation comparisons with other state-of-the-art improved algorithms were conducted on the CEC2017 and CEC2022 test sets. Statistical tests, convergence analysis, and stability analysis show that the AMBWO exhibits a superior overall performance. Finally, the applicability and superiority of the AMBWO was further verified by several engineering optimization problems.

## 1. Introduction

Optimization problems exist in various real-life fields [1], including engineering, medicine, economics, management, and agriculture. We need to make the best decisions with limited resources and specific constraints. In order to solve these problems, we have developed many artificial intelligence techniques, including neural network techniques, deep learning techniques, and optimization techniques [2]. Optimization techniques are methods and tools used to find the optimal solution to a problem [3]. These methods are widely used in various fields and aim to find the best solution or to make the best decision under given conditions and constraints to finally achieve a specific goal [4]. These optimization methods can be classified into deterministic and stochastic algorithms. Deterministic algorithms are good at handling linear, continuous, differentiable, and convex problems, but they encounter challenges when facing real-world nonlinear, nonconvex, high-dimensional, and non-differentiable problems, especially those characterized by discrete search spaces [5,6]. On the contrary, metaheuristic algorithms have shown significant efficacy in solving these problems. Metaheuristic algorithms employ a stochastic approach throughout the problem-solving process to generate a feasible solution space, systematically explore solutions within that space, evaluate the individual fitness using a fitness function, and make adjustments to achieve an optimal solution [7]. Over the past few decades, metaheuristic algorithms have been applied across various fields with notable success, including UAV path planning [8,9,10], the wireless sensor network coverage optimization problem [11,12], image segmentation [13,14], the traveling salesman problem [15,16], the energy supply design optimization problem [17], and feature selection problems [18,19]. The optimization process in metaheuristic algorithms can usually be divided into two phases: exploration and exploitation. Exploration searches the entire feasible space, while exploitation focuses on refining the best solution within a specific region. A well-designed algorithm should strike a balance between these two phases [20]. Metaheuristic algorithms can be categorized into four main groups based on their sources of inspiration: evolution-based algorithms (EAs), physics-based algorithms (PAs), human-based algorithms (HAs), and swarm-based algorithms (SAs) [21].

EAs are a class of metaheuristic algorithms inspired by natural evolutionary principles. The Genetic Algorithm (GA) [22] is one of the widely used evolutionary algorithms, proposed by Holland based on Darwinian evolutionary theory. Evolutionary Strategies (ESs) [23] and Differential Evolution (DE) [24] also belong to the category of evolution-based algorithms. Physics-based algorithms are usually inspired by physical phenomena, and one of the most classical examples is Simulated Annealing [25], which was proposed based on the principle of solid-state annealing in metallurgy by Metropolis et al. Other physics-based algorithms include the Multi-Verse Optimizer (MVO) [26], the Gravitational Search Algorithm (GSA) [27], the Equilibrium Optimizer (EO) [28], the Kepler Optimization Algorithm (KOA) [29], and Nuclear Reaction Optimization (NRO) [30]. Human-based algorithms are usually inspired by human behavior. Early research in this category includes Harmony Search (HS) [31], which mimics musicians’ improvisations to achieve optimization. Other algorithms in this group include Teaching–Learning-Based Optimization (TLBO) [32], Social Evolution and Learning Optimization (SELO) [33], the Political Optimizer (PO) [34], and the Chef-Based Optimization Algorithm (CBOA) [35]. Swarm-based algorithms draw inspiration from the social behavior of different populations of organisms in natural environments. Particle Swarm Optimization (PSO) [36], developed by Eberhart and Kennedy, is a well-known swarm-based metaheuristic inspired by the foraging behavior of birds. It conceptualizes birds as particles (objective function) looking for food in a forest-like search space. During each iteration, the motion of each particle considers its own optimal solution and the optimal solution obtained by the group, which ultimately guides the particle to converge to the location with the most food (the optimal solution). Many other noteworthy algorithms have emerged in this category in the last decades, including Artificial Bee Colony (ABC) [37], the Whale Optimization Algorithm (WOA) [38], Dwarf Mongoose Optimization (DMO) [39], Tuna Swarm Optimization (TSO) [40], the African Vulture Optimization Algorithm (AVOA) [41], the Marine Predators Algorithm (MPA) [42], and the Genghis Khan Shark Optimizer (GKSO) [43]. These algorithms provide diverse and flexible tools for solving optimization problems by modeling collective behavior in nature.

The beluga whale optimization (BWO) algorithm is an optimization method inspired by the group hunting habits of beluga whales [44]. It aims to build optimization models by mimicking their swimming, hunting, and whale falling behaviors. These three behaviors correspond to different search phases in the BWO algorithm: the exploration phase, the exploitation phase, and the whale fall phase. BWO has been used to solve a number of complex optimization problems, including path planning [45,46], energy management [47,48], and machine learning parameter optimization [49,50]. Chen et al. incorporate stochastic reverse learning and Gaussian variation strategies into BWO. Li et al. use Sobel sequences to initialize the population and then use optimal domain perturbation to improve the quality of the population. To enhance the global optimization capability of BWO, Li et al. introduced circular chaotic mapping and the Cauchy variation strategy. The above literature attempts to enhance the performance of BWO. However, it still faces challenges, such as imbalanced exploration and exploitation, insufficient population diversity, slow convergence, susceptibility to local optima, and low convergence accuracy. 

To address these challenges, we propose an augmented multi-strategy beluga whale optimization called AMBWO. In AMBWO, we first introduce the adaptive population learning strategy for improving the exploration capability of BWO, which increases the adaptability of the algorithm by fully learning the effective information of the population. In the exploitation phase, we introduce a roulette equilibrium selection strategy to facilitate the balance between exploitation and exploration and reduce the risk of falling into local optima. In addition, we introduce an adaptive avoidance strategy to ensure the diversity of the population so that the algorithm has faster convergence and better stability. In this study, the effectiveness of the AMBWO algorithm was tested by comparing it with many state-of-the-art improved methods on 41 test functions from the CEC2017 test set and the CEC2022 test set. The experimental data were analyzed using the statistical methods of the Friedman test and Wilcoxon rank-sum test. The experimental results show that the AMBWO algorithm produced better results than the basic BWO algorithm and its competitors. In addition to this, we applied AMBWO to solve some engineering design optimization problems in an efficient approach. In summary, the main work of this study is as follows:

(1) In the exploration phase, the adaptive population learning strategy is introduced with the aim of enhancing the global exploration capability of BWO by absorbing effective information from the superior population;

(2) In the exploitation phase, the roulette equilibrium selection strategy is used to balance the exploitation and exploration capabilities of BWO and enhance the adaptability of the algorithm;

(3) The adaptive avoidance strategy offers more quality candidate agents for BWO, enhancing the population diversity and reducing the probability of the BWO falling into a local optimum;

(4) The performance of the AMBWO was comprehensively evaluated using the CEC2017 and CEC2022 test suites and several constrained engineering problems.

The remaining sections of this paper are organized as follows: A concise overview of the BWO algorithm is provided in Section 2, entitled “Mathematical Model of BWO”. In Section 3, titled “The Proposed AMBWO”, we present three improvement strategies employed in AMBWO. Section 4 presents the results of experiments conducted on the CEC2017 test set, CEC2022 test set, and several engineering problems. Finally, Section 5 concludes the paper with a summary and outlook.

## 2. Mathematical Model of BWO

In this section, we present the mathematical model of the BWO algorithm in preparation for the next section on enhancing the BWO. The BWO algorithm consists of four phases: an initialization phase, an exploration phase, an exploitation phase, and a whale fall phase. The details of each of them are shown separately in the following section.

### 2.1. Initialization Phase

BWO is a swarm-based metaheuristic algorithm that employs randomly initialized beluga whales (denoted as X) as search agents. The search agents form a matrix with N rows and D columns, where N denotes the number of candidate agents and D denotes the spatial dimension of the problem, as shown in Equation (1). In BWO, the range of the X is determined by the upper (UB) and lower (LB) limits of the problem. The matrix (X) consists of the position vectors of the candidate population, where each position vector is randomly generated using Equation (2):

(1) $$\begin{array}{c} X=\left[\begin{array}{c} X_{1}\\ X_{2}\\ \vdots \\ X_{i}\\ \vdots \\ X_{N} \end{array}\right]=\left[\begin{array}{cccccc} x_{1,1} & x_{1,2} & \cdots & x_{1,j} & \cdots & x_{1,D}\\ x_{2,1} & x_{2,2} & \cdots & x_{2,j} & \cdots & x_{2,D}\\ \vdots & \vdots & \ddots & \vdots & \ddots & \vdots \\ x_{i,1} & x_{i,2} & \cdots & x_{i,j} & \cdots & x_{i,D}\\ \vdots & \vdots & \ddots & \vdots & \ddots & \vdots \\ x_{N,1} & x_{N,2} & \cdots & x_{N,j} & \cdots & x_{N,D} \end{array}\right] \end{array}$$

(2) $$\begin{array}{c} X_{i}=LB+rand\left(1,D\right)\times \left(UB-LB\right) \end{array},i=1,2,\ldots N$$

where i=1,2,…,N is the number of beluga whales, and j=1,2,…,dim is the dimension of the beluga whales.

### 2.2. Exploration Phase

The exploration phase of BWO is based on extensive research on swimming behavior. Beluga whales are paired with other beluga whales for synchronized or mirrored movements. The mathematical model is expressed as the following equation:

(3) $$\left\{\begin{array}{l} X_{i,j}^{t+1}=X_{i,p_{j}}^{t}+\left(X_{random,p_{1}}^{t}-X_{i,p_{j}}^{t}\right)\left(1+r_{1}\right)\sin\left(2*pi*r_{2}\right),j=even\\ X_{i,j}^{t+1}=X_{i,p_{j}}^{t}+\left(X_{random,p_{1}}^{t}-X_{i,p_{j}}^{t}\right)\left(1+r_{1}\right)\cos\left(2*pi*r_{2}\right),j=odd \end{array}\right.$$

where t is the current iteration. $X_{i,j}^{t}$ is the current position of the $i^{th}$ beluga whale in the $j^{th}$ dimension. $X_{i,j}^{t+1}$ is the new position obtained by $X_{i,j}^{t}$. $p_{j}\in [1,\dim]$ and random∈[1,N] are integers. $X_{i,p_{j}}^{t}$ denotes the position of the $i^{th}$ beluga whale in the $p_{j}^{th}$ dimension. $r_{1},r_{2}\in rand\left(0,1\right)$ are random numbers. The BWO will choose which motion mode to follow depending on whether the selection dimension is odd or even.

### 2.3. Exploitation Phase

During the exploitation phase, beluga whales actively send their location information to their partners. We can observe cooperative foraging and movement among the beluga whales, and the phenomenon can be described as the following mathematical equation:

(4) $$X_{i}^{t+1}=r_{3}\times X_{best}^{t}-r_{4}\times X_{i}^{t}+C_{1}\times LF\times \left(X_{random}^{t}-X_{i}^{t}\right)$$

(5) $$C_{1}=2\times r_{4}\times \left(1-t/T\right)$$

where $X_{best}^{t}$ is the location of the beluga whale with the smallest fitness value in the current iteration (t). $r_{3},r_{4}\in rand\left(0,1\right)$ are random numbers. T is the maximum iterations. LF is the value of the Levy flight, and the mathematical formulas for the LF are shown below:

(6) $$\sigma ={\left(\frac{\Gamma (1+\beta )\times \sin(\pi \beta /2)}{\Gamma ((1+\beta )/2)\times \beta \times 2^{(\beta -1)/2}}\right)}^{1/\beta }$$

(7) $$LF=0.05\times \frac{\mu \times \sigma }{{\left|v\right|}^{1/\beta }}$$

where μ and v are random numbers obeying a Gaussian distribution with a β constant equal to 1.5. The transition between the exploration phase and exploitation phase is adaptively executed based on the balance factor ($B_{f}$). The mathematical model is expressed as follows:

(8) $$B_{f}=B*\left(1-t/T\right)$$

where B∈rand(0,1). Based on the value of the $B_{f}$, the beluga shifts between the exploration and exploitation phases. When $B_{f}\geq 0.5$, the exploration phase happens, whereas the exploitation phase is contingent upon $B_{f}<0.5$, and as the number of iterations increases, the range of the $B_{f}$ moves from (0,1) to (0,0.5) in a nonlinear fashion, indicating the significant change in the probabilities for the exploitation and exploration phases.

### 2.4. Whale Fall Phase

Beluga whales also encounter predators in the ocean and eventually die. In BWO, an equation is applied to update the locations of dead beluga whales, which can be described as follows:

(9) $$X_{i}^{t+1}=r_{5}\times X_{i}^{t}-r_{6}\times X_{random}^{t}+r_{7}\times X_{step}$$

(10) $$X_{step}=\left(UB-LB\right)\times e^{\left(-C_{2}\times t/T\right)}$$

(11) $$C_{2}=2\times W_{f}\times N$$

(12) $$W_{f}=0.1-0.05\times t/T$$

where $r_{5},r_{6},r_{7}\in rand(0,1)$. The factor $C_{2}$ is associated with the likelihood of a whale fall and the size of the population. $W_{f}$ is the probability of the whale fall factor, which decreases nonlinearly from 0.1 to 0.05 as the number of iterations increases, indicating a gradual decrease in the probability of a whale fall as the distance between the beluga whales and the food source diminishes.

## 3. The Proposed AMBWO

From Section 2, it can be seen that the BWO algorithm fails to utilize the population information well in the exploration phase and lacks sufficient global exploration capability. In the exploitation phase, the BWO guides the local search by adopting the current optimal search agent, which lacks sufficient adaptability and suffers from the problems of falling into local optima and low convergence accuracy. To address these problems, this section proposes three improvement strategies to enhance the overall performance of the BWO algorithm. First, an adaptive population learning strategy is proposed to enhance the exploration capability. Second, a roulette equilibrium selection strategy is proposed to balance the exploration and exploitation performance of the BWO. Finally, at the end of each iteration, an adaptive avoidance strategy is presented to assist the algorithm in eliminating the local optimum and improving the population quality. The details of these strategies are explained in the following subsections.

### 3.1. Adaptive Population Learning Strategy

The BWO failed to show a sufficient global search capability in the exploration phase. To address this deficiency, this paper proposes the adaptive population learning strategy (APLS). The APLS consists of two elements. Firstly, a correction is made for the original search strategy. We introduce an adaptive perturbation factor to increase the stochasticity of the algorithm, expand the search range, and help discover more candidate solutions. Furthermore, we introduce a population-based Gaussian learning mechanism to fully utilize the high-quality population to improve the solution quality. The mathematical model of the APLS is specified as follows:

(13) $$E_{factor}=\left(E_{\max}-E_{\min}\right)\times e^{\left(t/T\right)}-E_{\min}$$

(14) $$\left\{\begin{array}{l} X_{i,j}^{t+1}=X_{i,p_{j}}^{t}+E_{factor}\times \left(r_{8}\times X_{random,p_{1}}^{t}-X_{i,p_{j}}^{t}\right)\left(1+r_{1}\right)\sin\left(2*pi*r_{2}\right),j=even\\ X_{i,j}^{t+1}=X_{i,p_{j}}^{t}+E_{factor}\times \left(r_{8}\times X_{random,p_{1}}^{t}-X_{i,p_{j}}^{t}\right)\left(1+r_{1}\right)\cos\left(2*pi*r_{2}\right),j=odd \end{array}\right.$$

(15) $$X_{mean}=\sum_{i=1}^{N/2}\omega_{i}\times X_{i}^{t}$$

(16) $$\omega_{i}=\frac{\ln\left(0.5N+1\right)-\ln\left(i\right)}{\sum_{i=1}^{0.5N}\left(\ln\left(0.5N+1\right)-\ln\left(i\right)\right)}$$

(17) $$Cov=\frac{\sum_{i=1}^{0.5N}\left(X_{i}^{t}-X_{mean}\right)\times {\left(X_{i}^{t}-X_{mean}\right)}^{†}}{0.5N-1}$$

(18) $$X_{i}^{t+1}=Gaussian\left(X_{mean},Cov\right)+r_{9}\times \left(X_{mean}-X_{i}^{t}\right)$$

where $E_{factor}$ is the exploration-phase factor, $E_{\min}=1.1$ is the initial exploration phase, and $E_{\max}=2.5$ is the final exploration phase. $r_{8},r_{9}\in rand(0,1)$. As the iteration number (t) increases, the nonlinear decrease in the $E_{factor}$ makes the initial solution’s weight in the solution gradually decrease, while the weight of the $\left(r_{8}\times X_{random,p_{1}}^{t}-X_{i,p_{j}}^{t}\right)\left(1+r_{1}\right)\sin\left(2*pi*r_{2}\right)$ part gradually increases, expanding the global search capability of the AMBWO in the exploration phase. $X_{mean}$ is obtained by weighting the individuals in the top half of the fitness rankings. † denotes transposed symbols. Each beluga whale chooses one of these two methods to perform the exploration behavior at each iteration.

### 3.2. Roulette Equilibrium Selection Strategy

BWO converges the population by learning from the optimal beluga during the exploitation phase. This approach can help the algorithm converge quickly, but moving blindly with reference to the optimal point will lead to a weakening of the diversity of the other individuals and ultimately to a local optimum. Although the best individual cannot be selected blindly, high-quality individuals are still the target for the remaining individuals to learn. In order to enhance the population diversity and retain some global exploration ability on the basis of a strong exploitation capability, this paper proposes a roulette equilibrium selection strategy (RESS):

(19) $$S_{i}=\alpha_{1}\times \frac{\left(Dis_{i}-Dis_{\min}\right)}{Dis_{\max}}+\left(1-\alpha_{1}\right)\times \frac{\left(Fit_{i}-Fit_{\min}\right)}{Fit_{\max}}$$

where $Dis_{i}$ denotes the Euclidean distance between $X_{i}$ and the optimal agent. $Dis_{\min}$ and $Dis_{\max}$ are the minimum and maximum distances in the population. $Fit_{i}$ denotes the fitness of $X_{i}$. $Fit_{\min}$ and $Fit_{\max}$ are the minimum and maximum fitness in the population. $\alpha_{1}$ is the weighting coefficient of the two evaluation metrics and, in this study, was set as 0.5. After the population update, the score (S) for each beluga whale was calculated using Equation (19), and then a reference individual ($X_{reference}^{t}$) was selected to replace the optimal individual using the roulette wheel principle. The modified exploitation strategy is represented as follows:

(20) $$X_{i}^{t+1}=r_{3}\times X_{reference}^{t}-r_{4}\times X_{i}^{t}+C_{1}\times LF\times \left(X_{random}^{t}-X_{i}^{t}\right)$$

### 3.3. Adaptive Avoidance Strategy

While beluga whales are predators, there are more advanced predators that attack them. Inspired by the Gazelle optimization algorithm, this paper introduces an adaptive avoidance strategy (AAS). This strategy can help the algorithm to eliminate the local optimality and improve the quality of the solution:

(21) $$X_{i}^{t+1}=\left\{\begin{array}{l} X_{i}^{t}+CF\times \left(LB+r_{10}\times \left(UB-LB\right)\right)\times U,rand\leq 0.34\\ X_{i}^{t}+\left(0.34\times (1-r_{11})+r_{11}\right)\times \left(X_{r1}^{t}-X_{r2}^{t}\right),rand>0.34 \end{array}\right.$$

(22) $$CF={\left(1-t/T\right)}^{\left(2-t/T\right)}$$

where $r_{9},r_{10}\in rand(0,1)$. U is the binary vector, which is constructed by generating a random number in the range [0, 1]. $X_{r1}^{t}$ and $X_{r2}^{t}$ are two random individuals in the beluga whales.

### 3.4. Procedure of AMBWO

**Figure 1:**
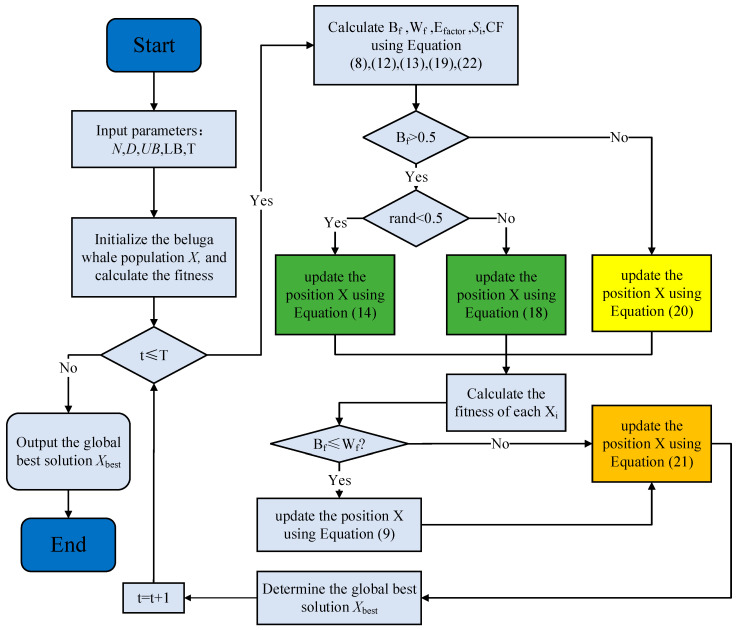
The flowchart of AMBWO.

Summarizing Section 3.1, Section 3.2 and Section 3.3, the flowchart and pseudocode of the AMBWO are shown in Figure 1 and Algorithm 1.
**Algorithm 1** CBBWO algorithmInitialize the size of search agents (the population of the beluga whales) N, and the position of each beluga whale X, set the initial iteration parameter t=1, and the max iteration times T. While t<T
  Calculate $B_{f}$, $W_{f}$, $E_{factor}$, $S_{i}$, CF using Equations (8), (12), (13), (19) and (22)
  For i=1 to N
   If $B_{f}>0.5$//Adaptive population learning strategy (APLS)
    If rand>0.5
    Update the position X using Equation (18)//$X_{i}^{t+1}=Gaussian\left(X_{mean},Cov\right)+r_{9}\times \left(X_{mean}-X_{i}^{t}\right)$
    Else
    Update the position X using Equation (14)//$\left\{\begin{array}{l} X_{i,j}^{t+1}=X_{i,p_{j}}^{t}+E_{factor}\times \left(r_{8}\times X_{random,p_{1}}^{t}-X_{i,p_{j}}^{t}\right)\left(1+r_{1}\right)\sin\left(2*pi*r_{2}\right),j=even\\ X_{i,j}^{t+1}=X_{i,p_{j}}^{t}+E_{factor}\times \left(r_{8}\times X_{random,p_{1}}^{t}-X_{i,p_{j}}^{t}\right)\left(1+r_{1}\right)\cos\left(2*pi*r_{2}\right),j=odd \end{array}\right.$
    End if
   Else//Roulette equilibrium selection strategy (RESS)
    Update the position X using Equation (20)//$X_{i}^{t+1}=r_{3}\times X_{reference}^{t}-r_{4}\times X_{i}^{t}+C_{1}\times LF\times \left(X_{random}^{t}-X_{i}^{t}\right)$
   End if
   If $B_{f}<W_{f}$
    Update the position X using Equation (9)//$X_{i}^{t+1}=r_{5}\times X_{i}^{t}-r_{6}\times X_{random}^{t}+r_{7}\times X_{step}$
   End if
   Update the position X using Equation (21)//$X_{i}^{t+1}=\left\{\begin{array}{l} X_{i}^{t}+CF\times \left(LB+r_{10}\times \left(UB-LB\right)\right)\times U,rand\leq 0.34\\ X_{i}^{t}+\left(0.34\times (1-r_{11})+r_{11}\right)\times \left(X_{r1}^{t}-X_{r2}^{t}\right),rand>0.34 \end{array}\right.$
  End for
  t=t+1
 End while
Output the optimal solution $X_{best}$.

### 3.5. Time Complexity Analysis

The time complexity of the algorithm is determined by the number of variable dimensions (D), the population size (N), and the number of iterations (T). The time complexity of the original BWO algorithm is determined by the initialization and update operators. The time complexity of the exploitation and exploration phases of the BWO is denoted as $O\left(T\times N\times D\right)$. The time complexity of the whale fall phase can be denoted as $O\left(T\times 0.1N\times D\right)$ according to the original literature. Therefore, the time complexity of the BWO is approximately evaluated as $O\left(T\times 1.1N\times D\right)$.

For the AMBWO, the APLS technique and the RESS technique did not increase the time complexity. Therefore, the time complexity of the exploration and exploitation phases of the AMBWO is $O\left(T\times N\times D\right)$. The combination of the AAS method and the whale fall phase still resulted in a time complexity of $O\left(T\times 0.1N\times D\right)$. In conclusion, the time complexity of the AMBWO is $O\left(T\times 1.1N\times D\right)$, which is unchanged compared to the BWO.

## 4. Experimental Evaluation

This section provides a comprehensive evaluation of the proposed AMBWO algorithm by going through several experiments. A total of 42 test functions were used to examine the performance of the AMBWO, of which 29 were taken from the CEC2017 test set and 12 were taken from the CEC2022 test set. Section 4.1 shows the details of these functions and presents the comparative algorithms that were involved in the experiments. In Section 4.2, we analyze the impact of each strategy on the AMBWO. Section 4.3 and Section 4.4 show the results of the comparison between the AMBWO and the competitors on the two test suites, respectively. Finally, the performance of the AMBWO is further validated by several engineering constraint problems in Section 4.5.

### 4.1. Experiment Setting

#### 4.1.1. Benchmark Suite Descriptions

The CEC2017 function suite contains a total of 29 different functions. The F2 function was excluded from the comparison due to its instability and in order to maintain the sequence alignment (i.e., F1–F29). The CEC2017 test suite consists of unimodal (two functions), multimodal (seven functions), hybrid (ten functions), and composite (ten functions) functions. The unimodal functions are mathematical challenges characterized by the presence of a critical point. More precisely, one point has both local and global properties. The multimodal functions are characterized by the presence of multiple critical points. In addition, the specified test functions include composite and hybrid problems. Hybrid functions can exhibit either multi-peak or single-peak behavior depending on their underlying purpose. Composite functions are formed by combining the rotation and translation functions of different functions. The CEC2022 contains a total of 12 functions consisting of single-peak (one function), basic (four functions), hybrid (three functions), and composite (four functions) functions. The search space for all the functions is defined as [−100, 100]. Since finding the optimal solution is a challenging mission due to the multimodal, hybrid, and composite structure of these functions, these two test sets were employed to evaluate the performance of the AMBWO. Table 1 and Table 2 present the specifics of the two test sets.

#### 4.1.2. Comparative Algorithms and Parameter Settings

In order to fully demonstrate the superiority of the algorithm proposed in this paper, seven state-of-the-art metaheuristic algorithms were utilized for comparison. These seven algorithms are all improved versions of basic algorithms, including HBWO-JS [51], FDBARO [52], MCOA [53], DETDO [54], BEESO [55], DTSMA [56], and IDE-EDA [57]. HBWO-JS is an enhanced version of the BWO algorithm. IDE-EDA is an improved DE variant. All the other improved algorithms are variants of well-known algorithms. For a fair comparison, the parameter settings of all the competitors were set according to the original literature, displayed in Table 3. The maximum number of iterations was 500, and the number of the population was 30.

All the experiments in this study were conducted on a computer with 48 GB RAM, running the Microsoft Windows 10 operating system. The CPU used was an AMD R9 9950X with a clock speed of 4.30 GHz. The experimental simulation process was implemented in MATLAB R2021b.

### 4.2. Effectiveness Analysis of the Strategy

In this subsection, we discuss the impacts of the proposed improvement strategies on AMBWO. Based on the control variable approach, six AMBWO variants are proposed, including AMBWO-1, AMBWO-2, AMBWO-3, AMBWO-12, AMBWO-23, and AMBWO-13. The first three variants each combine a single improvement strategy, and the latter three variants combine two improvement strategies separately, as shown in Table 4.

**Figure 2:**
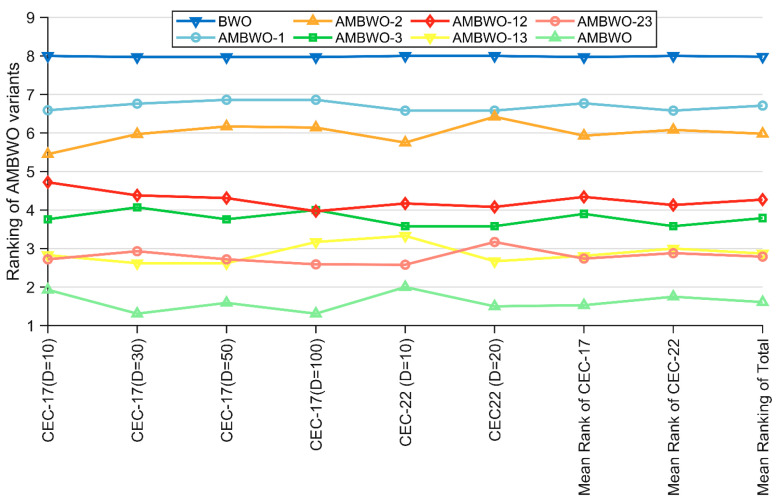
Ranking of AMBWO and six variants based on the Friedman test.

The algorithms mentioned in Table 4 were run thirty times on the CEC2017 and CEC2022 test sets, and the results obtained were analyzed using the Friedman test. The rankings are recorded in Table 5 and visualized in Figure 2. Based on the *p*-values in the last column of Table 5, we can conclude that there is a performance difference between the BWO and these six AMBWO variants as well as the AMBWO. The specific analysis is as follows. All three AMBWO variants combining a single improvement strategy are ranked better than the basic BWO, which indicates that all three strategies can improve the performance of the BWO separately. Based on the rankings of these three variants, we can learn that the impacts of the three improvement strategies on the BWO are, in descending order, ASS > RESS > APLS. Similarly, according to the rankings of AMBWO-12, AMBWO-13, and AMBWO-23, the ASS has the greatest impact on BWO, with the RESS and APLS reducing the impact in that order. It is worth noting that the ranking of the AMBWO combining the three improved strategies is the best among all the algorithms, which shows that the impacts of the three improved strategies on BWO do not cancel each other but rather promote each other to further enhance the capability of BWO.

### 4.3. Results and Analysis for CEC2017 Test Suite

In this subsection, we provide a comprehensive assessment of the AMBWO’s performance for the CEC2017 test set. Since the CEC2017 test set has four dimensions and the experimental data are relatively large, we record the mean and standard deviation obtained from the experiments in Table A1, Table A2, Table A3 and Table A4 in Appendix A. The Wilcoxon rank-sum test and Friedman test will be applied to analyze these data to evaluate the performance of the AMBWO from a statistical perspective. Additionally, the results of the convergence and robustness analysis of the AMBWO will be presented following the statistical tests.

**Figure 3:**
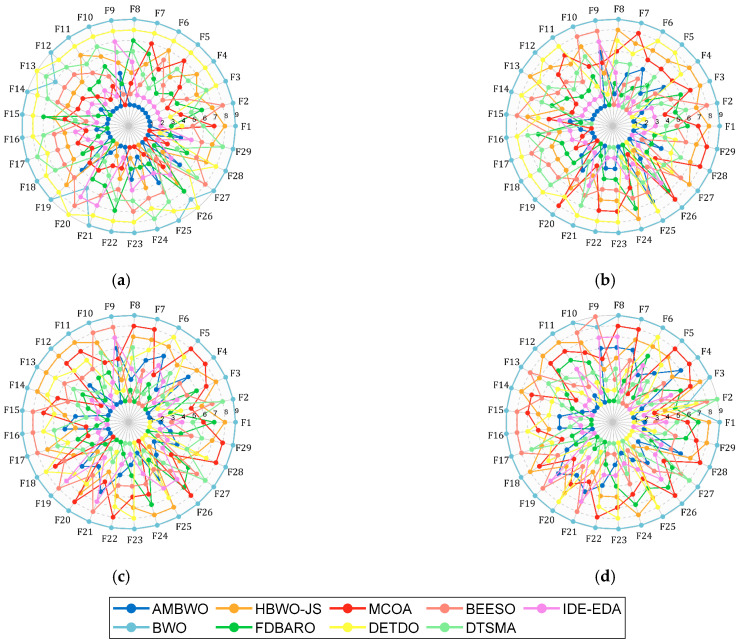
Ranking of AMBWO and competitors based on CEC2017. (**a**) D = 10; (**b**) D = 30; (**c**) D = 50; (**d**) D = 100.

First of all, Figure 3 shows a spider plot based on the ranking of each algorithm on each function to briefly demonstrate the performance of the AMBWO. We can tentatively conclude that the AMBWO has an excellent performance. The specific analysis is carried out below.

To comprehensively demonstrate the superiority of the proposed algorithm, we will use the Wilcoxon rank-sum test to verify whether the results of each run of the AMBWO differ significantly from the other algorithms at a significance level of *p* = 0.05. The null hypothesis (H0) is that there is no significant difference between the two algorithms. When *p* < 0.05, we reject the null hypothesis, indicating a significant difference between the two algorithms. When *p* > 0.05, we accept the null hypothesis, indicating no significant difference between the two algorithms (i.e., similar algorithm performances). 

**Figure 4:**
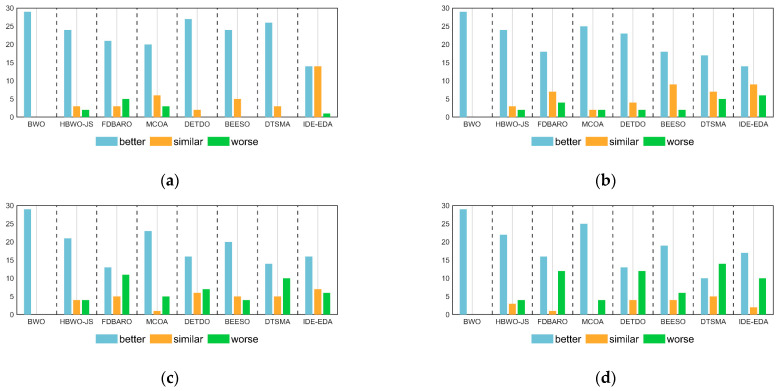
The visualization of Wilcoxon rank-sum test results for CEC2017. (**a**) D = 10; (**b**) D = 30; (**c**) D = 50; (**d**) D = 100.

Table 6 shows the Wilcoxon test results of the AMBWO and the competitors for 10D, 30D, 50D, and 100D of the CEC2017 test functions. The symbols “+”, “−”, and “=” indicate that the AMBWO performed better than, worse than, and similar to the comparison algorithms, respectively. According to Table 6, the number of “+” symbols is more than the total number of “−” symbols, which indicates that the AMBWO outperformed the competing algorithms in different dimensions of the CEC2017. Figure 4 visualizes the Wilcoxon rank-sum test results for the AMBWO and the comparison algorithms. 

When D = 10, the AMBWO is better (worse) than the BWO, HBWO-JS, FDBARO, MCOA, DETDO, BEESO, DTSMA, and IDE-EDA in the 29(0), 24(2), 21(5), 20(3), 27(0), 24(5), 26(0), and 14(1) functions. Thus, the AMBWO is the best algorithm among these advanced methods in solving the 10D CEC2017 test function.

When D = 30, the AMBWO is better (worse) than the BWO, HBWO-JS, FDBARO, MCOA, DETDO, BEESO, DTSMA, and IDE-EDA in the 29(0), 24(2), 18(4), 25(2), 23(2), 18(2), 17(5), and 14(6) functions. Thus, the AMBWO is the best algorithm among these advanced methods in solving the 30D CEC2017 test function.

When D = 50, the AMBWO is better (worse) than the BWO, HBWO-JS, FDBARO, MCOA, DETDO, BEESO, DTSMA, and IDE-EDA in the 29(0), 21(4), 13(11), 23(5), 16(7), 20(4), 14(10), and 17(10) functions. Thus, the AMBWO is the best algorithm among these advanced methods in solving the 50D CEC2017 test function.

When D = 100, the AMBWO is better (worse) than the BWO, HBWO-JS, FDBARO, MCOA, DETDO, BEESO, DTSMA, and IDE-EDA in the 29(0), 22(4), 16(12), 25(4), 13(12), 19(6), 10(14), and 17(10) functions. Thus, the DTSMA is the best algorithm among these advanced methods in solving the 100D CEC2017 test function.

**Figure 5:**
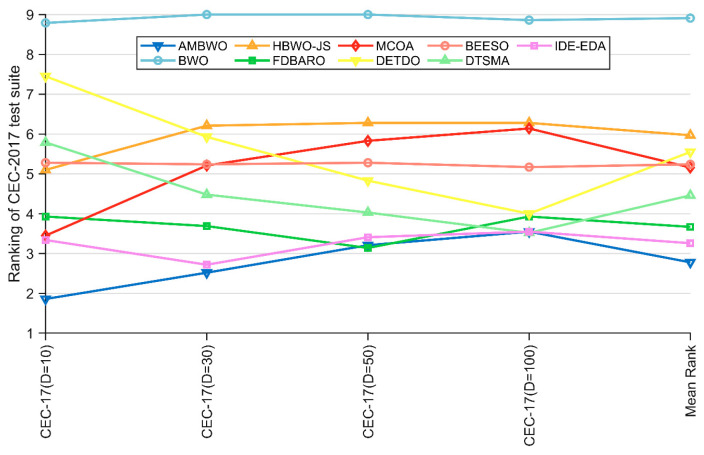
The visualization of Friedman test results for CEC2017.

Except for the Wilcoxon rank-sum test, the Friedman test that appears in Section 4.2 was also employed to evaluate the experimental results of the AMBWO and comparison algorithms. The results of the test are recorded in Table 7 and visualized in Figure 5. According to the last row of Table 7, the *p*-value is less than 0.05 in all cases, so we can conclude that there is a difference between the AMBWO and the other compared algorithms. According to Figure 5, we can learn that the AMBWO ranks first in 10D and 30D and after the FDBARO and DTSMA in 50D and 100D, respectively. Although the AMBWO failed to achieve the best ranking in all the dimensions, its overall performance was the best among all the algorithms, achieving an average ranking of 2.78. According to the Friedman test results, the AMBWO performed better in low dimensions and similar to the best algorithm tested in high dimensions. The FDBARO, which performed best in 50D, showed little fluctuation in its performance in the other dimensions. The best performer at 100D, the DTSMA, is the one whose ranking increases as the dimensions increase.

**Figure 6:**
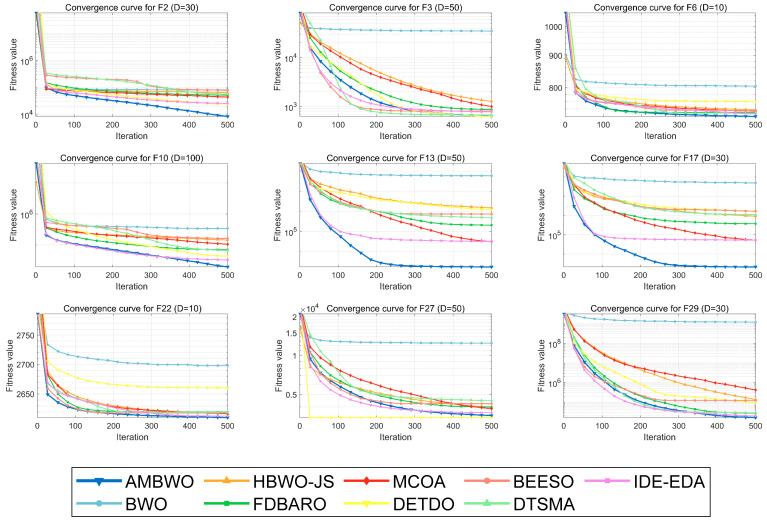
The convergence curves of AMBWO and competitors for CEC2017.

**Figure 7:**
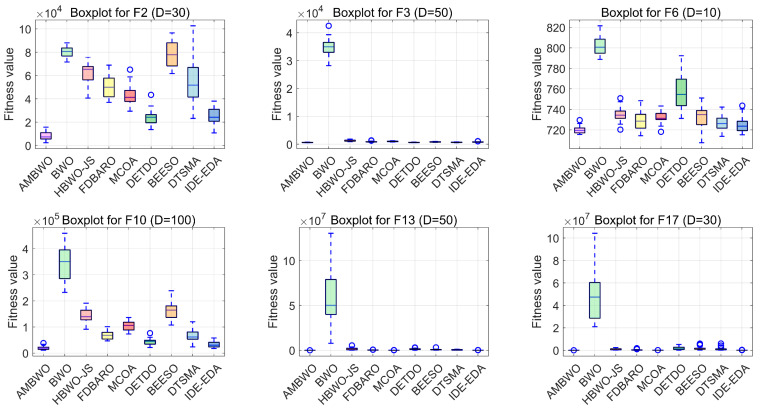

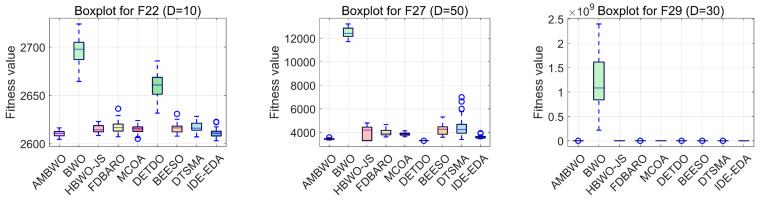
The boxplots of the AMBWO and competitors for CEC2017.

**Figure A1:**
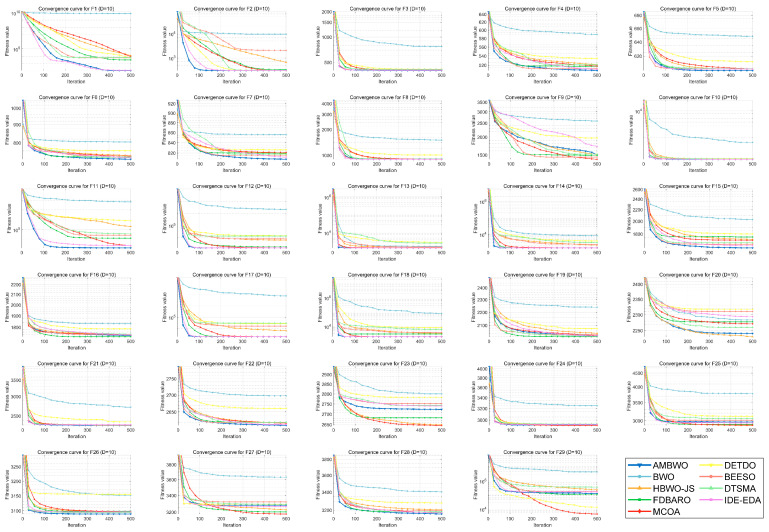
The convergence curves of AMBWO and other competitors based on CEC2017 (D = 10).

**Figure A2:**
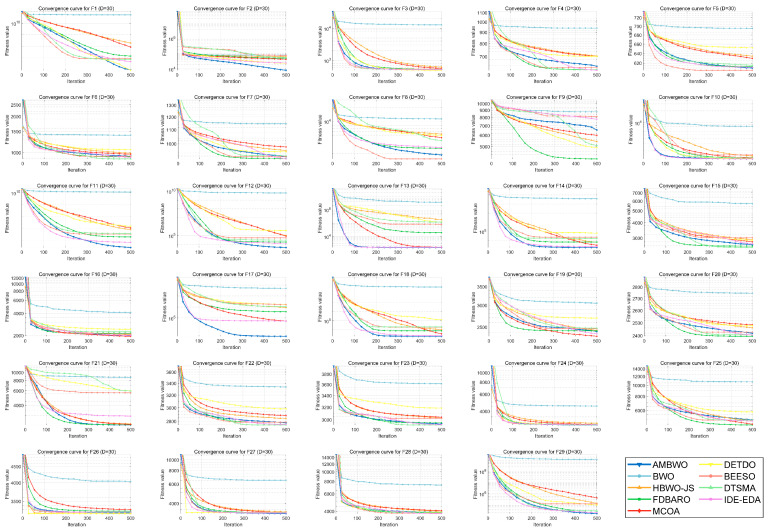
The convergence curves of AMBWO and other competitors based on CEC2017 (D = 30).

**Figure A3:**
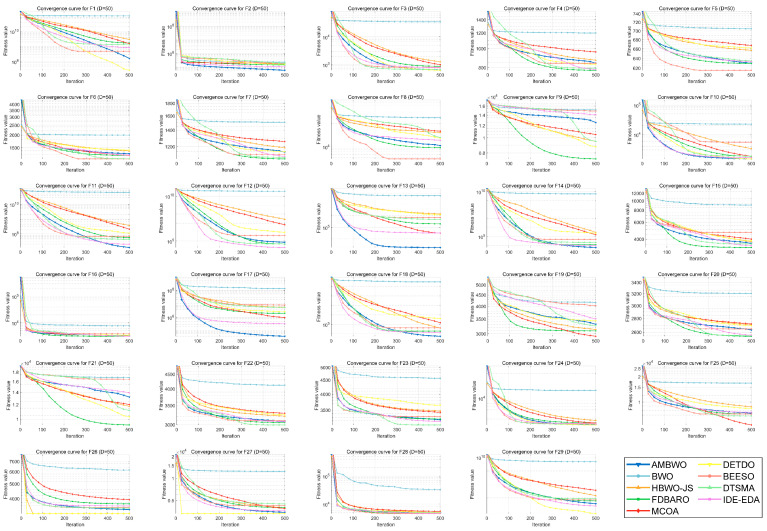
The convergence curves of AMBWO and other competitors based on CEC2017 (D = 50).

**Figure A4:**
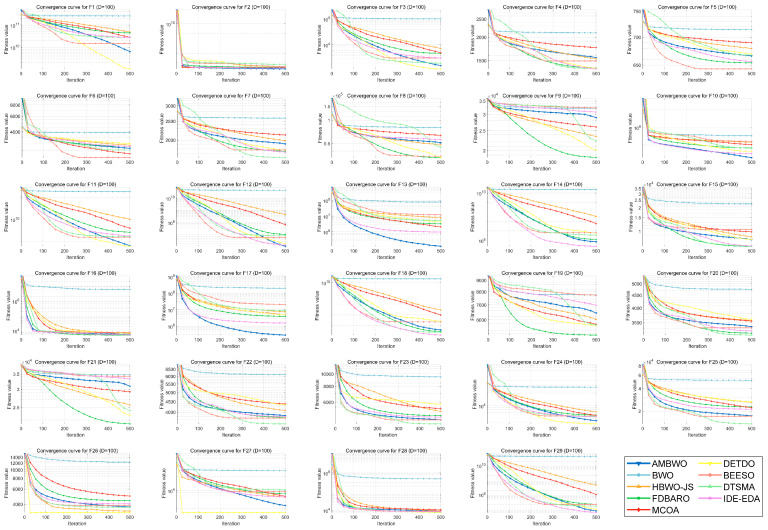
The convergence curves of AMBWO and other competitors based on CEC2017 (D = 100).

**Figure A5:**
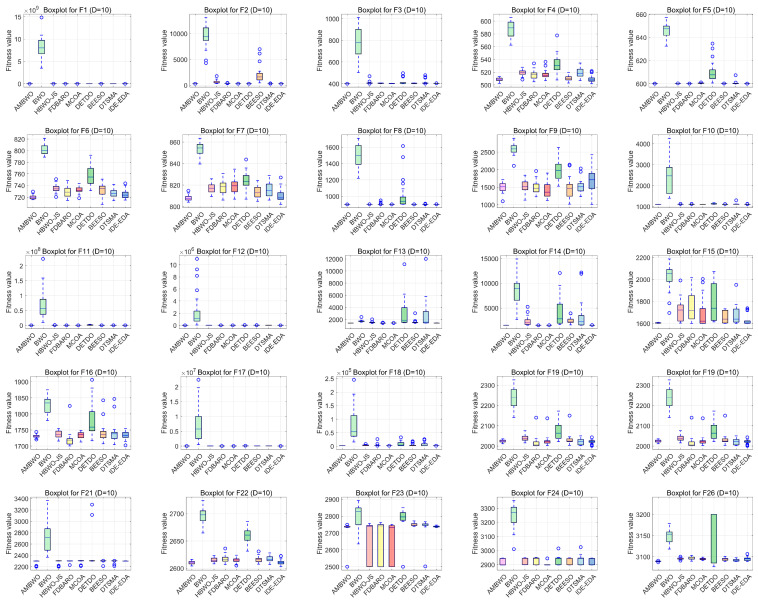

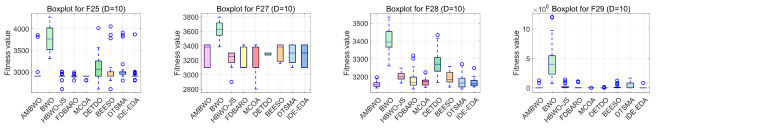
The boxplots of AMBWO and other competitors based on CEC2017 (D = 10).

**Figure A6:**
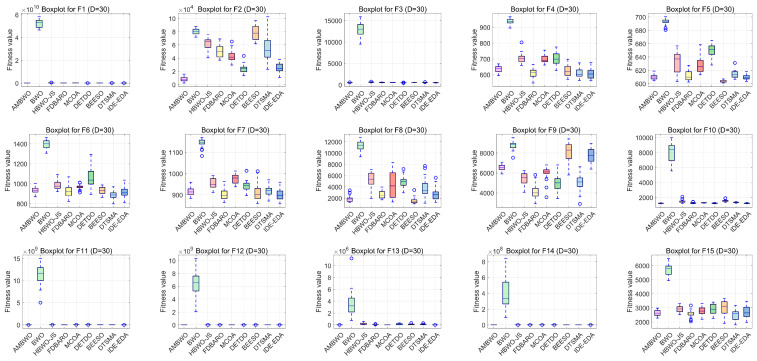

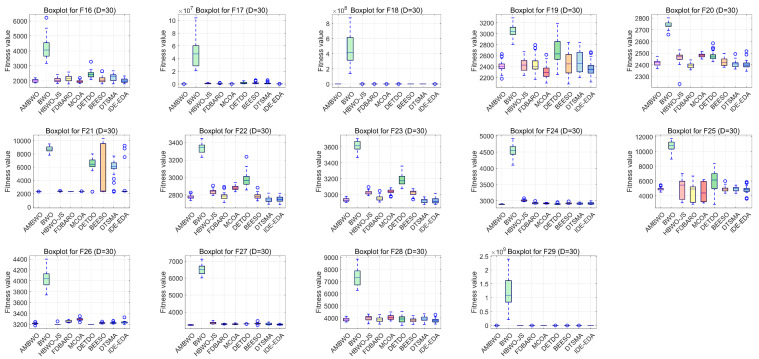
The boxplots of AMBWO and other competitors based on CEC2017 (D = 30).

**Figure A7:**
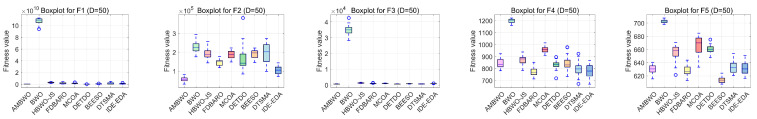

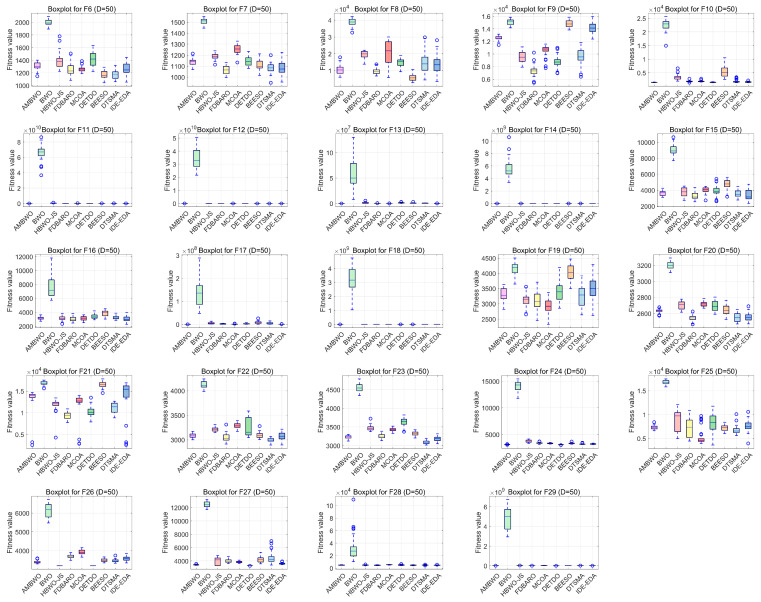
The boxplots of AMBWO and other competitors based on CEC2017 (D = 50).

**Figure A8:**
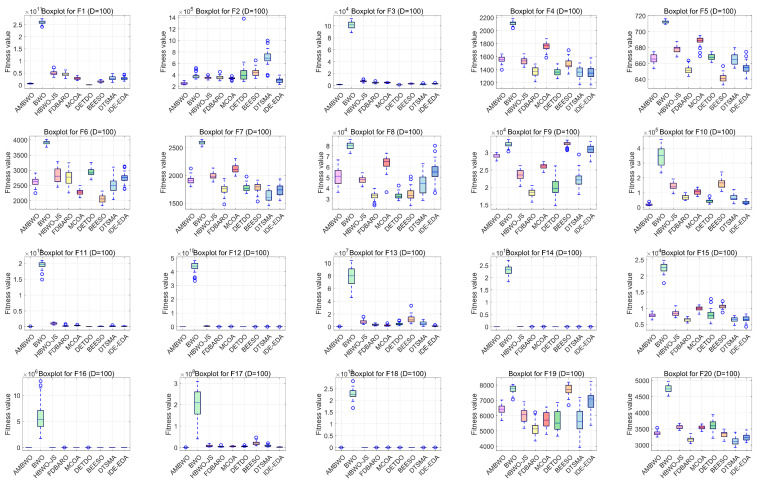

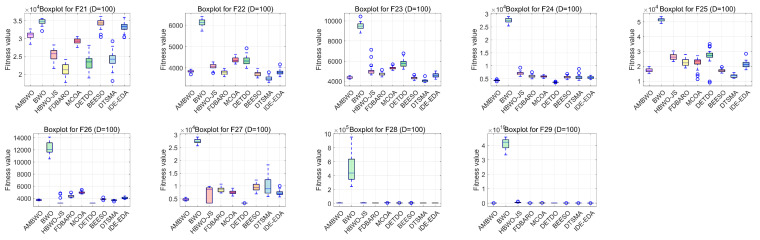
The boxplots of AMBWO and other competitors based on CEC2017 (D = 100).

A convergence analysis and stability analysis of the AMBWO on the CEC2017 test set is performed in this segment. Figure 6 and Figure 7 show the convergence curves and box plots for different classes of partial functions in the CEC2017 test set. The complete convergence curves and box plots are displayed in Figure A1, Figure A2, Figure A3, Figure A4, Figure A5, Figure A6, Figure A7 and Figure A8 in Appendix B. In F2, the AMBWO was able to exploit the problem space consistently, obtaining the best convergence values. This is because the APLS strategy enhances the exploitation of the AMBWO. F3 and F6 are multimodal functions, and the AMBWO possesses a better convergence accuracy for F3 and faster convergence and a better convergence accuracy for F6. This is due to the fact that the RESS and ASS enhance the exploration capability of AMBWO as well as the proper switching between exploitation and exploration. AMBWO is able to discover new search regions and switch from exploitation to exploration at the right time. For F10, F13, and F17, the AMBWO achieved the fastest convergence speed and highest convergence accuracy. For F22 and F29, the AMBWO had the best convergence performance of all the algorithms. For F27, the AMBWO was worse than the DETDO but still ranked second. Based on the convergence curves, we confirm that the APLS, RESS, and ASS substantially improve the convergence performance of AMBWO.

Boxplots are useful tools for visualizing quartile-based statistics. They allowed us to analyze the distribution of the data and compare the performance of AMBWO with those of other methods. In Figure 7, the tentacles of each box plot represent the minimum and maximum values obtained by a single method, while the edges of the rectangular boxes indicate the lower and higher quartiles for each algorithm. Circles indicate outliers. The AMBWO shows the best performance with no outliers for F2, F3, and F22. For F6, F10, and F27, the AMBWO has bad values, but its boxes are compact, showing a more concentrated distribution of solutions. For F13, F17, and F29, the AMBWO and IDE-EDA are comparable in terms of their stabilities. The APLS strategy plays an important role in improving the performance by leveraging information from dominant populations to guide individual evolution and enhance stability. The RESS achieves a balance between exploitation and exploration, and the ASS improves the quality of the solutions to minimize significant fluctuations in the fitness values throughout the iterations. 

Finally, in order to assess the scalability of the proposed AMBWO method, the performances of the AMBWO and BWO in different dimensions are further analyzed. Based on the experimental results presented in Figure 4, it is clear that the proposed AMBWO method outperformed the original BWO algorithm, whether it was 10D, 30D, 50D, or 100D. The proposed AMBWO method outperformed the original BWO algorithm in terms of finding the ideal solution of various functions, regardless of their complexity. Therefore, it can be inferred that the AMBWO method proposed in this paper has better optimization capabilities and greatly enhanced stability compared to the original BWO algorithm.

### 4.4. Results and Analysis for CEC2022 Test Suite

**Figure 8:**
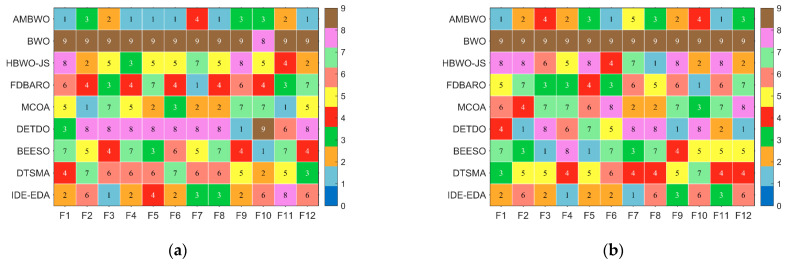
The ranking of AMBWO and competitors for CEC2022. (**a**) D = 10, (**b**) D = 20.

In this subsection, we discuss the results of the AMBWO based on the CEC2022 test set. In order to fully evaluate the performance of the proposed algorithm, we conducted experiments in 10 and 20 dimensions. The experimental environment and parameter settings were the same as those in Section 4.3. Table A5 and Table A6 in Appendix A record the test results of these algorithms run independently 30 times in both dimensions, including the means and standard deviations. Figure 8 illustrates the rankings of the AMBWO and the competitors for each function based on the mean value. We can find that the AMBWO has the best overall performance. The experimental data will be analyzed next using the Wilcoxon rank-sum test and the Friedman test.

**Figure 9:**
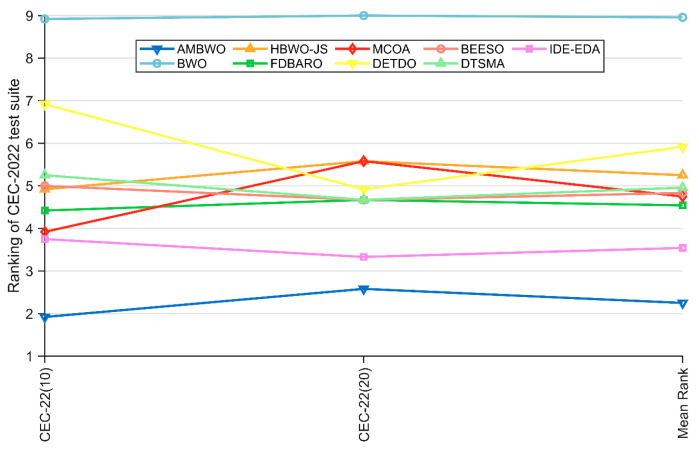
The Friedman scores of AMBWO and competitors for CEC2022.

**Figure 10:**
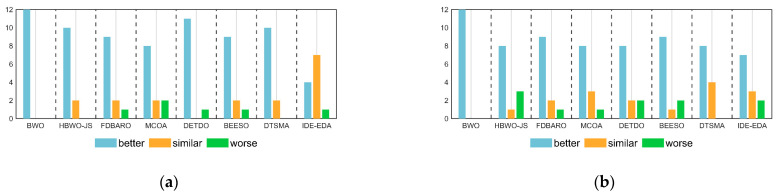
The visualization of the Wilcoxon rank-sum test results for CEC2022. (**a**) D = 10, (**b**) D = 20.

Table 8 summarizes the results of the Wilcoxon rank-sum test and Friedman test for the AMBWO and competitors under the CEC2022 10D and 20D test functions. For the Friedman test, the *p*-values in Table 8 indicate that there is a significant difference between the AMBWO and the other algorithms. The AMBWO is ranked first in both dimensions and the BWO is ranked last. Figure 9 displays the Friedman test scores for the AMBWO and the competitors. According to Figure 9, the scores of the AMBWO do not fluctuate much, which indicates its good scalability. The whole curve of the AMBWO is minimized at every point, which indicates that its overall performance was the best. Figure 10 visualizes the Wilcoxon rank-sum test results for the AMBWO and the comparison algorithms for the CEC2022. 

When D = 10, the AMBWO is better (worse) than the BWO, HBWO-JS, FDBARO, MCOA, DETDO, BEESO, DTSMA, and IDE-EDA in the 12(0), 10(2), 9(1), 8(2), 11(1), 8(1), 10(0), and 4(1) functions. Thus, the AMBWO is the best algorithm among these advanced methods in solving the 10D CEC2022 test function.

When D = 20, the AMBWO is better (worse) than the BWO, HBWO-JS, FDBARO, MCOA, DETDO, BEESO, DTSMA, and IDE-EDA in the 12(0), 8(3), 9(1), 8(1), 8(2), 9(2), 8(0), and 7(2) functions. Thus, the AMBWO is the best algorithm among these advanced methods in solving the 20D CEC2022 test function.

### 4.5. Results and Analysis of Engineering Problems

Following the experiments and analysis in Section 4.1, Section 4.2, Section 4.3 and Section 4.4, we clearly observe the superior optimization performance of the AMBWO in terms of the test functions. However, the primary objective of metaheuristic algorithms is to address real-world problems. Therefore, in this subsection, we further validate the effectiveness and applicability of AMBWO through its application to practical problems. To assess the practical applicability and scalability of the proposed algorithm, we apply it to three typical real-world engineering problems. Furthermore, to highlight the superiority of AMBWO, we continue to compare its results with the optimization outcomes of the seven advanced algorithms mentioned earlier in Section 4.1.2.

#### 4.5.1. Tension/Compression Spring Design Problem

**Figure 11:**
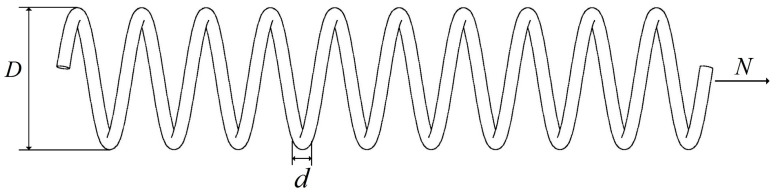
Problem with tension compression spring design.

This design problem aims to minimize the weight of a stretch/compression spring by finding three crucial parameters of the spring, including the wire diameter (d), coil diameter (D), and number of coils (N). The structure of this engineering problem is illustrated in Figure 11, and the mathematical model for the spring design optimization problem is as follows: 

Variables:

(23) $$x=\left[x_{1},\;x_{2},\;x_{3}]=[d,\;D,\;N\right]$$

Minimize:

(24) $$f\left(x\right)=\left(x_{3}+2\right)x_{2}x_{1}^{2}$$

Subject to:

(25) $$\begin{array}{l} g_{1}\left(x\right)=1-\frac{x_{2}^{3}x_{3}}{71785x_{1}^{4}}\leq 0,\\ g_{2}\left(x\right)=\frac{4x_{2}^{2}-x_{1}x_{2}}{12566\left(x_{2}x_{1}^{3}-x_{1}^{4}\right)}+\frac{1}{5108x_{1}^{2}}\leq 0,\\ g_{3}\left(x\right)=1-\frac{140.45x_{1}}{x_{2}^{2}x_{3}}\leq 0,\\ g_{4}\left(x\right)=\frac{x_{1}+x_{2}}{1.5}-1\leq 0 \end{array}$$

With bounds:

(26) $$0.05\leq x_{1}\leq 2.00,\;0.25\leq x_{2}\leq 1.30,\;2.00\leq x_{3}\leq 15.0$$

The experimental results of the AMBWO and eight different comparative algorithms are shown in Table 9. It is evident from the table that the AGOA’s optimization results outperform those of the other comparative algorithms, with a value of 0.012669. At this optimal solution, the design variable values for the spring are *x* = [0.051231, 0.34579, 11.95991].

#### 4.5.2. Pressure Vessel Design Problem

**Figure 12:**
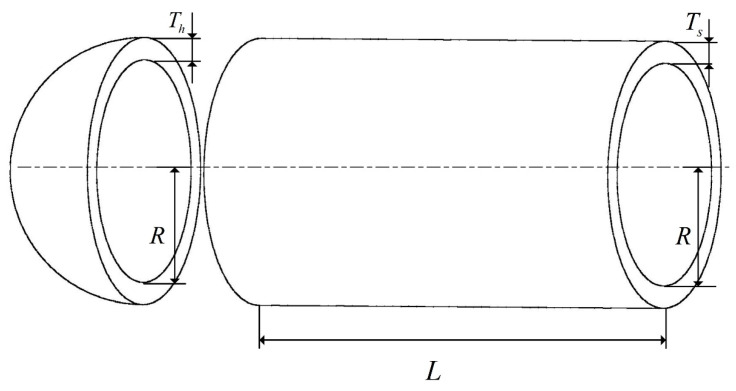
Problem with pressure vessel design.

The structure of the pressure vessel design problem is illustrated in Figure 12. The design objective of this problem is to minimize the costs while meeting the usage requirements. The four optimization parameters include the container thickness (Ts), head thickness (Th), inner radius (R), and head length (L). The mathematical representation of this problem is detailed below.

Variables:

(27) $$x=\left[x_{1},\;x_{2},\;x_{3},x_{4}]=[T_{s},\;T_{h},\;R,L\right]$$

Minimize:

(28) $$f(x)=0.6224x_{1}x_{3}x_{4}+1.7781x_{2}x_{3}^{2}+3.1661x_{1}^{2}x_{4}+19.84x_{1}^{2}x_{3}$$

Subject to:

(29) $$\begin{array}{l} g_{1}(x)=x_{1}+0.0193x_{3}\leq 0\\ g_{2}(x)=x_{2}+0.00954x_{3}\leq 0\\ g_{3}(x)=-\pi x_{3}^{2}x_{4}+\frac{4}{3}\pi x_{3}^{3}+1296000\leq 0\\ g_{4}(x)=x_{4}-240\leq 0 \end{array}$$

With bounds:

(30) $$1\leq x_{1},x_{2}\leq 99,10\leq x_{3},x_{4}\leq 200$$

Like the other compared problems, the proposed algorithm achieved a superior performance in this problem. The detailed results are provided in Table 10. In Table 10, the AMBWO shows the best performance in solving this problem. The AMBWO algorithm obtained the lowest objective function value at 5885. 3328 through its unique search mechanism and optimization strategy, and it better balances the abilities of the global and local searches.

#### 4.5.3. Three-Bar Truss Design Problem

**Figure 13:**
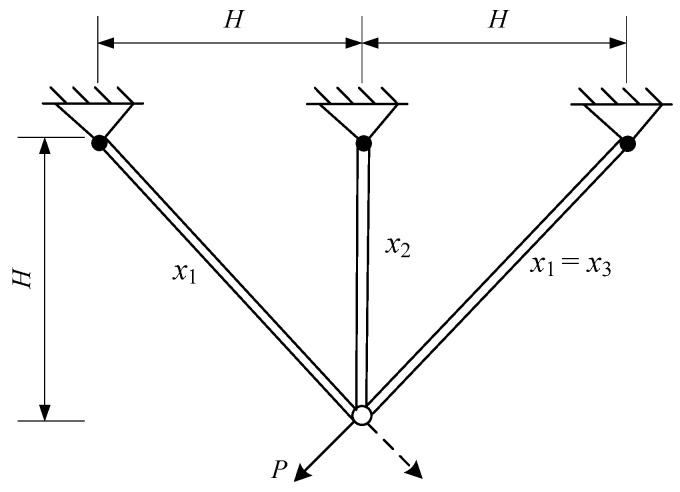
Problem with three-bar truss design.

The optimization design of a three-bar truss refers to minimizing the mass of the truss while satisfying stress and deformation constraints. The design variables for this optimization problem are the cross-sectional areas of the left and right bars, denoted as x_1_, and the cross-sectional area of the middle bar, denoted as x_2_, as shown in Figure 13. The mathematical model for this optimization design problem is as follows:

Variables:

(31) $$x=\left[\begin{array}{c} x_{1},\;x_{2} \end{array}\right]$$

Minimize:

(32) $$f\left(x\right)=\left(2\sqrt{2x_{1}}+x_{2}\right)\times L$$

Subject to:

(33) $$\begin{array}{l} g_{1}(x)=\frac{\sqrt{2}x_{1}+x_{2}}{\sqrt{2}x_{1}^{2}+2x_{1}x_{2}}P-\sigma \leq 0\\ g_{2}(x)=\frac{x_{2}}{\sqrt{2}x_{1}^{2}+2x_{1}x_{2}}P-\sigma \leq 0\\ g_{3}(x)=\frac{1}{x_{1}+\sqrt{2}x_{2}}P-\sigma \leq 0 \end{array}$$

With bounds:

(34) $$0\leq x_{1}\leq 1,\;0\leq x_{2}\leq 1$$

where

(35) $$L=100\mathrm{cm},\;P=2\;kN\cdot {\mathrm{cm}}^{-2},\;\sigma =2\;kN\cdot {\mathrm{cm}}^{-2}$$

The three-bar truss problem was solved by the AMBWO, and the result is given in Table 11. The results obtained by the other competitors are also outlined in Table 10. As shown in Table 10, compared with nine optimization techniques, the AMBWO obtained the minimum volume for the three-bar truss design problem with a value of 262.8958. At this optimal point, the values of the design variables are x = [0.7887, 0.4082].

## 5. Conclusions

In this paper, we propose an augmented multi-strategy beluga whale optimization called AMBWO. AMBWO applies three techniques to enhance the performance, including an adaptive population learning strategy, a roulette equilibrium selection strategy, and an adaptive avoidance strategy. These strategies enable AMBWO to effectively avoid local optima and smoothly transition between the exploration and exploitation phases. To validate the effectiveness of AMBWO, it was evaluated using the benchmark test suites CEC2017 and CEC2022. In addition, various visual depictions of convergence curves and boxplots are provided, which help to clearly show the excellent performance statistics of AMBWO. The results show the significant benefits of AMBWO in different dimensions of the CEC2017 and CEC2022 benchmark functions. In order to validate the algorithm’s ability to solve real-world problems, it was tested by applying it to three engineering optimization problems. The results show that AMBWO performed well on both unconstrained and constrained problems. Compared with other algorithms, AMBWO consistently obtained the optimal solutions in these problems, showing its robustness and wide adaptability. From the results, AMBWO has achieved some success, but there are still some deficiencies that need to be further addressed. AMBWO needs to be mined even further to improve the poor performance in some of the functions. The APLS strategy is sensitive to the number of populations, and it is necessary to further investigate this sensitivity. As a perspective of this work, we intend to extend the use of the AMBWO algorithm in the future, such as to UAV tasking, feature selection, cloud resource management, and computer vision. In addition, the development of multi-objective and binary variants of AMBWO will be considered as future work to solve increasingly complex optimization problems in real-world environments.

## Figures and Tables

**Table 1:** CEC2017 test functions.

Type	ID	Range	Dim
Unimodal	F1–F2	[−100,100]	[10,30,50,100]
Multimodal	F3–F9	[−100,100]	[10,30,50,100]
Hybrid	F10–F19	[−100,100]	[10,30,50,100]
Composition	F20–F29	[−100,100]	[10,30,50,100]

**Table 2:** CEC2022 test functions.

Type	ID	Range	Dim
Unimodal	F1	[−100,100]	[10,20]
Basic	F2–F5	[−100,100]	[10,20]
Hybrid	F6–F8	[−100,100]	[10,20]
Composition	F9–F12	[−100,100]	[10,20]

**Table 3:** Parameter settings for different comparative algorithms.

Algorithm	Setting Values
AMBWO	$W_{f}=[0.05,0.1]$
BWO	$W_{f}=[0.05,0.1]$
HBWO-JS	$W_{f}=[0.05,0.1]$
FDBARO	k=1
MCOA	$F_{0}=0.5,CR=0.9$
DETDO	$F_{0}=0.9$
BEESO	$TR=0.25,TR_{2}=0.6,C_{1}=0.5,C_{2}=0.05,C_{3}=2$
DTSMA	*z* = 0.03, *q* = 0.9
IDE-EDA	k=3,H=5,τ=0.9

**Table 4:** Description of the AMBWO variants.

Strategy	BWO	AMBWO-1	AMBWO-2	AMBWO-3	AMBWO-12	AMBWO-13	AMBWO-23	AMBWO
APLS	No	Yes	No	No	Yes	Yes	No	Yes
RESS	No	No	Yes	No	Yes	No	Yes	Yes
ASS	No	No	No	Yes	No	Yes	Yes	Yes

**Table 5:** The Friedman test results obtained by AMBWO variants.

Function	Algorithm	BWO	AMBWO-1	AMBWO-2	AMBWO-3	AMBWO-12	AMBWO-13	AMBWO-23	AMBWO	*p*-Value
CEC-17	D = 10	8.00	6.59	5.45	3.76	4.72	2.83	2.72	1.93	9.93E-29
	D = 30	7.97	6.76	5.97	4.07	4.38	2.62	2.93	1.31	8.65E-34
	D = 50	7.97	6.86	6.17	3.76	4.31	2.62	2.72	1.59	2.55E-34
	D = 100	7.97	6.86	6.14	4.00	3.97	3.17	2.59	1.31	1.38E-34
CEC-22	D = 10	8.00	6.58	5.75	3.58	4.17	3.33	2.58	2.00	1.06E-10
	D = 20	8.00	6.58	6.42	3.58	4.08	2.67	3.17	1.50	1.00E-12
Mean rank of CEC-17		7.97	6.77	5.93	3.90	4.34	2.81	2.74	1.53	N/A
Mean rank of CEC-22		8.00	6.58	6.08	3.58	4.13	3.00	2.88	1.75	N/A
Mean rank of total		7.98	6.71	5.98	3.79	4.27	2.87	2.79	1.61	N/A

**Table 6:** The Wilcoxon rank-sum test results obtained by AMBWO and comparison algorithms.

AMBWO vs. +/=/−	CEC-17 Test Suite			
	10D	30D	50D	100D
BWO	29/0/0	29/0/0	29/0/0	29/0/0
HBWO-JS	24/3/2	24/3/2	21/4/4	22/3/4
FDBARO	21/3/5	18/7/4	13/5/11	16/1/12
MCOA	20/6/3	25/2/2	23/1/5	25/0/4
DETDO	27/2/0	23/4/2	16/6/7	13/4/12
BEESO	24/5/0	18/9/2	20/5/4	19/4/6
DTSMA	26/3/0	17/7/5	14/5/10	10/5/14
IDE-EDA	14/14/1	14/9/6	16/7/6	17/2/10

**Table 7:** The Friedman test results obtained by the AMBWO and comparison algorithms.

Algorithm	CEC-17 Test Suite				
	10D	30D	50D	100D	Mean Rank
AMBWO	1.86	2.52	3.21	3.55	2.78
BWO	8.79	9.00	9.00	8.86	8.91
HBWO-JS	5.10	6.21	6.28	6.28	5.97
FDBARO	3.93	3.69	3.14	3.93	3.67
MCOA	3.45	5.21	5.83	6.14	5.16
DETDO	7.45	5.93	4.83	4.00	5.55
BEESO	5.28	5.24	5.28	5.17	5.24
DTSMA	5.79	4.48	4.03	3.52	4.46
IDE-EDA	3.34	2.72	3.41	3.55	3.26
*p*-value	3.54E-27	8.85E-23	3.16E-20	1.59E-18	N/A

**Table 8:** The results of the Friedman test and Wilcoxon rank-sum test obtained by AMBWO and comparison algorithms.

CEC-22 Test Suite					
Algorithm	10D		20D		Mean rank
AMBWO	1.92	N/A	2.58	N/A	2.25
BWO	8.92	12/0/0	9.00	12/0/0	8.96
HBWO-JS	4.92	10/2/0	5.58	8/1/3	5.25
FDBARO	4.42	9/2/1	4.67	9/2/1	4.54
MCOA	3.92	8/2/2	5.58	8/3/1	4.75
DETDO	6.92	11/0/1	4.92	8/2/2	5.92
BEESO	5.00	9/2/1	4.67	9/1/2	4.83
DTSMA	5.25	10/2/0	4.67	8/4/0	4.96
IDE-EDA	3.75	4/7/1	3.33	7/3/2	3.54
*p*-value	3.04E-08	N/A	2.06E-06	N/A	N/A

**Table 9:** The results of comparison for the tension compression spring design issue.

Algorithm	*d*	*D*	*N*	*F(x)*
BWO	0.05	0.317199	14.06477	0.012739
HBWO-JS	0.052348	0.372345	10.44257	0.012696
FDBARO	0.057568	0.515351	5.761796	0.013257
MCOA	0.050044	0.318407	13.94698	0.012716
DETDO	0.059481	0.574776	4.732177	0.01369
BEESO	0.055105	0.444587	7.53203	0.012868
DTSMA	0.055464	0.45382	7.344742	0.013046
IDE-EDA	0.05137	0.349034	11.76998	0.012683
AMBWO	0.051231	0.34579	11.95991	0.012669

**Table 10:** The results of comparison for the pressure vessel design issue.

Algorithm	*Ts*	*Th*	*R*	*L*	*F(x)*
BWO	0.818673	0.441278	41.67	190.8592	6373.9791
HBWO-JS	0.99700	0.492821	51.6584	85.7092	6374.4572
FDBARO	1.09049	0.545982	56.5015	53.8861	6701.6314
MCOA	12.96419	7.150134	42.09829	176.6392	6059.7489
DETDO	1.25885	0.622249	65.2252	10	7319.0006
BEESO	1.00924	0.49887	52.2924	81.138	6409.2154
DTSMA	13.18886	7.349468	42.09791	176.6464	6059.8523
IDE-EDA	12.96996	7.337754	42.03656	177.4064	6067.2991
AMBWO	0.77816	0.384649	40.31962	200	5885.3328

**Table 11:** The results of comparison for the pressure vessel design issue.

Algorithm	X1	X2	*F(x)*
BWO	0.79134	0.40105	263.9007
HBWO-JS	0.58959	0.20568	263.8994
FDBARO	0.78784	0.41062	263.8966
MCOA	0.78980	0.40507	263.8974
DETDO	0.78853	0.40871	263.9020
BEESO	0.79409	0.39313	263.9170
DTSMA	0.78863	0.40835	263.8958
IDE-EDA	0.78712	0.41265	263.8986
AMBWO	0.7887	0.4082	262.8958

## Data Availability

Data will be made available upon request.

## Appendix A

**Table A1:** Statistical measures of AMBWO with other algorithms using CEC2017, D = 10.

Function No.	Index	AMBWO	BWO	HBWO-JS	FDBARO	MCOA	DETDO	BEESO	DTSMA	IDE-EDA
F1	Mean	1.0012E+02	8.1054E+09	1.2061E+04	3.0022E+03	8.6869E+03	5.3130E+03	5.7985E+03	6.5307E+03	1.0931E+02
	Std	3.5878E-01	2.4360E+09	1.2727E+04	4.0306E+03	5.7169E+03	3.5992E+03	6.3478E+03	4.0277E+03	1.9888E+01
F2	Mean	3.0000E+02	9.4717E+03	6.5374E+02	3.2566E+02	3.0127E+02	3.0077E+02	2.0193E+03	3.0607E+02	3.0000E+02
	Std	3.3482E-08	2.0152E+03	3.2875E+02	4.9234E+01	2.8242E+00	1.5989E+00	1.5051E+03	1.8320E+01	1.3219E-03
F3	Mean	4.0000E+02	7.6902E+02	4.0822E+02	4.0413E+02	4.0233E+02	4.1366E+02	4.0438E+02	4.1121E+02	4.0134E+02
	Std	5.3822E-03	1.4381E+02	1.2097E+01	1.9624E+00	1.1220E+00	2.2753E+01	1.8150E+00	1.8612E+01	1.1937E+00
F4	Mean	5.0877E+02	5.8727E+02	5.1937E+02	5.1573E+02	5.1634E+02	5.3260E+02	5.1065E+02	5.1893E+02	5.0941E+02
	Std	2.6376E+00	1.2949E+01	4.6552E+00	6.3737E+00	5.9801E+00	1.3442E+01	4.1784E+00	7.5137E+00	5.3323E+00
F5	Mean	6.0001E+02	6.4614E+02	6.0007E+02	6.0003E+02	6.0051E+02	6.0995E+02	6.0006E+02	6.0046E+02	6.0001E+02
	Std	2.6382E-02	6.2357E+00	6.0021E-02	4.2322E-02	2.3716E-01	8.1629E+00	5.9777E-02	1.3164E+00	2.6105E-02
F6	Mean	7.1987E+02	8.0241E+02	7.3517E+02	7.2854E+02	7.3229E+02	7.5823E+02	7.3337E+02	7.2696E+02	7.2473E+02
	Std	3.3112E+00	8.4810E+00	6.4956E+00	8.3755E+00	5.3838E+00	1.6505E+01	9.9743E+00	6.7194E+00	7.2373E+00
F7	Mean	8.0814E+02	8.5388E+02	8.1687E+02	8.1788E+02	8.1902E+02	8.2404E+02	8.1340E+02	8.1589E+02	8.0999E+02
	Std	2.5209E+00	6.0131E+00	4.3050E+00	6.1631E+00	6.9735E+00	7.9520E+00	5.3419E+00	6.4195E+00	5.6307E+00
F8	Mean	9.0002E+02	1.5005E+03	9.0033E+02	9.0337E+02	9.0009E+02	1.0041E+03	9.0006E+02	9.0082E+02	9.0041E+02
	Std	8.2948E-02	1.4008E+02	3.7444E-01	1.0325E+01	1.4831E-01	1.7143E+02	1.8875E-01	2.0293E+00	5.5036E-01
F9	Mean	1.5042E+03	2.5867E+03	1.5078E+03	1.4875E+03	1.3990E+03	1.9673E+03	1.4500E+03	1.5175E+03	1.7209E+03
	Std	1.3741E+02	1.4987E+02	1.7289E+02	1.7391E+02	2.0596E+02	2.8551E+02	2.7031E+02	1.7333E+02	3.5235E+02
F10	Mean	1.1025E+03	2.3743E+03	1.1112E+03	1.1087E+03	1.1075E+03	1.1306E+03	1.1075E+03	1.1173E+03	1.1057E+03
	Std	1.3624E+00	7.6077E+02	5.0537E+00	5.3358E+00	1.5372E+00	1.8908E+01	3.7779E+00	3.3923E+01	5.0932E+00
F11	Mean	1.3585E+03	6.7411E+07	2.0613E+05	1.3549E+04	2.3419E+03	7.8953E+05	2.6517E+04	3.7194E+04	2.4101E+03
	Std	1.2044E+02	4.6841E+07	2.5496E+05	1.3282E+04	4.6919E+02	7.8064E+05	2.5225E+04	4.2188E+04	1.2732E+03
F12	Mean	1.3097E+03	2.2503E+06	5.8822E+03	1.6132E+03	1.3397E+03	1.3708E+04	7.3670E+03	1.2911E+04	1.3159E+03
	Std	2.8644E+00	2.7763E+06	3.4793E+03	1.3456E+03	1.6304E+01	9.9179E+03	6.9953E+03	1.2147E+04	1.3243E+01
F13	Mean	1.4135E+03	1.7292E+03	1.4987E+03	1.4125E+03	1.4298E+03	2.8967E+03	1.5743E+03	2.6131E+03	1.4143E+03
	Std	6.4963E+00	1.5536E+02	1.0697E+02	2.1271E+01	3.0731E+00	2.1833E+03	2.8448E+02	2.1211E+03	1.0633E+01
F14	Mean	1.5012E+03	8.4273E+03	2.2914E+03	1.5040E+03	1.5122E+03	3.9388E+03	2.3850E+03	3.1852E+03	1.5047E+03
	Std	6.2453E-01	2.9203E+03	8.4272E+02	2.4511E+00	3.3720E+00	2.6504E+03	5.8727E+02	2.6715E+03	8.0974E+00
F15	Mean	1.6046E+03	2.0263E+03	1.7187E+03	1.7536E+03	1.7078E+03	1.7990E+03	1.6517E+03	1.6723E+03	1.6327E+03
	Std	2.5736E+00	1.0404E+02	1.0223E+02	1.2401E+02	1.3736E+02	1.4938E+02	5.0701E+01	7.7196E+01	5.4069E+01
F16	Mean	1.7311E+03	1.8293E+03	1.7370E+03	1.7196E+03	1.7341E+03	1.7845E+03	1.7396E+03	1.7407E+03	1.7342E+03
	Std	4.8607E+00	2.5447E+01	1.1069E+01	2.1851E+01	9.6599E+00	5.2505E+01	2.5417E+01	2.8077E+01	1.1737E+01
F17	Mean	1.8069E+03	7.5029E+06	6.1979E+03	1.8166E+03	1.8341E+03	2.5822E+04	1.5723E+04	2.8829E+04	1.8208E+03
	Std	7.2559E+00	6.3447E+06	3.2339E+03	3.5692E+01	8.1653E+00	2.1201E+04	7.8876E+03	1.5023E+04	1.8020E+01
F18	Mean	1.9018E+03	8.0277E+04	3.3113E+03	2.9422E+03	1.9061E+03	8.1248E+03	3.7667E+03	6.1700E+03	1.9028E+03
	Std	8.2732E-01	5.7868E+04	1.5594E+03	4.5717E+03	2.3377E+00	7.7515E+03	3.9534E+03	6.1531E+03	3.6583E+00
F19	Mean	2.0238E+03	2.2383E+03	2.0389E+03	2.0143E+03	2.0234E+03	2.0735E+03	2.0305E+03	2.0221E+03	2.0203E+03
	Std	6.0987E+00	4.9683E+01	1.5017E+01	2.5596E+01	2.3117E+01	4.4098E+01	2.4834E+01	1.3217E+01	9.8362E+00
F20	Mean	2.2407E+03	2.2817E+03	2.2284E+03	2.2751E+03	2.2691E+03	2.3158E+03	2.3087E+03	2.2581E+03	2.2922E+03
	Std	5.3899E+01	3.4498E+01	4.7238E+01	5.7609E+01	5.7341E+01	5.3738E+01	2.8065E+01	6.0953E+01	4.1918E+01
F21	Mean	2.2883E+03	2.7137E+03	2.2904E+03	2.2996E+03	2.2970E+03	2.3630E+03	2.2997E+03	2.2978E+03	2.3006E+03
	Std	3.2639E+01	2.4935E+02	3.3052E+01	1.3327E+01	2.6747E+01	2.2975E+02	1.6754E+01	2.0014E+01	4.2948E-01
F22	Mean	2.6105E+03	2.6959E+03	2.6151E+03	2.6178E+03	2.6150E+03	2.6583E+03	2.6164E+03	2.6171E+03	2.6111E+03
	Std	3.3732E+00	1.5393E+01	4.2284E+00	6.7057E+00	4.4416E+00	1.3779E+01	5.4818E+00	5.5577E+00	5.1712E+00
F23	Mean	2.7230E+03	2.7983E+03	2.6471E+03	2.6804E+03	2.6441E+03	2.7812E+03	2.7502E+03	2.7409E+03	2.7384E+03
	Std	6.0715E+01	6.6531E+01	1.2099E+02	1.1082E+02	1.1964E+02	8.0185E+01	7.6385E+00	4.5797E+01	3.8978E+00
F24	Mean	2.9149E+03	3.2503E+03	2.9256E+03	2.9240E+03	2.9088E+03	2.9308E+03	2.9264E+03	2.9312E+03	2.9242E+03
	Std	2.2601E+01	7.6966E+01	2.2905E+01	2.4284E+01	1.9703E+01	2.7451E+01	2.3100E+01	3.4958E+01	2.2959E+01
F25	Mean	2.9655E+03	3.7738E+03	2.8802E+03	2.9013E+03	2.8872E+03	3.1110E+03	3.0090E+03	3.0551E+03	2.9376E+03
	Std	2.3667E+02	2.8586E+02	8.9494E+01	3.4937E+01	3.3666E+01	2.8714E+02	3.2170E+02	2.8064E+02	1.8195E+02
F26	Mean	3.0893E+03	3.1497E+03	3.0956E+03	3.0961E+03	3.0944E+03	3.1533E+03	3.0943E+03	3.0921E+03	3.0946E+03
	Std	6.0517E-01	1.5477E+01	2.0652E+00	3.6166E+00	2.1048E+00	5.8275E+01	3.3279E+00	2.6369E+00	4.0518E+00
F27	Mean	3.2812E+03	3.6291E+03	3.2259E+03	3.2008E+03	3.1764E+03	3.2909E+03	3.3182E+03	3.2920E+03	3.2642E+03
	Std	1.4238E+02	1.0760E+02	9.2354E+01	1.3364E+02	1.5346E+02	1.2671E+01	1.1083E+02	1.2289E+02	1.4294E+02
F28	Mean	3.1582E+03	3.4019E+03	3.2022E+03	3.1801E+03	3.1706E+03	3.2762E+03	3.1962E+03	3.1749E+03	3.1683E+03
	Std	1.5721E+01	6.8204E+01	1.9508E+01	4.4766E+01	2.0967E+01	6.8906E+01	3.3655E+01	3.8608E+01	2.3804E+01
F29	Mean	1.2684E+05	4.3603E+06	2.3803E+05	1.1060E+05	4.5187E+03	1.3660E+04	1.7717E+05	3.6222E+05	1.6710E+05
	Std	3.2733E+05	3.0166E+06	3.3003E+05	3.1008E+05	1.6901E+03	3.2150E+04	3.3522E+05	5.0826E+05	3.3233E+05

**Table A2:** Statistical measures of AMBWO with other algorithms using CEC2017, D = 30.

Function No.	Index	AMBWO	BWO	HBWO-JS	FDBARO	MCOA	DETDO	BEESO	DTSMA	IDE-EDA
F1	Mean	8.8336E+05	5.2691E+10	1.9299E+08	1.4126E+07	8.4307E+07	8.3133E+05	8.8395E+06	7.2400E+06	4.7152E+06
	Std	4.8613E+05	3.4239E+09	1.2528E+08	6.4590E+06	3.0705E+07	4.9314E+05	5.1253E+06	1.8604E+07	5.0445E+06
F2	Mean	8.2216E+03	8.0307E+04	6.3197E+04	5.1203E+04	4.3285E+04	2.3883E+04	7.7623E+04	5.5366E+04	2.5058E+04
	Std	3.4363E+03	4.7212E+03	8.5211E+03	9.3052E+03	8.2750E+03	6.0426E+03	1.0878E+04	1.7915E+04	6.9818E+03
F3	Mean	4.9761E+02	1.2841E+04	6.1777E+02	5.3149E+02	5.5806E+02	4.8716E+02	5.2953E+02	5.2032E+02	5.0670E+02
	Std	2.3500E+01	1.6206E+03	5.0968E+01	4.0940E+01	2.1870E+01	2.0077E+01	2.0883E+01	3.5402E+01	4.6372E+01
F4	Mean	6.3424E+02	9.3769E+02	7.0306E+02	6.1102E+02	6.9974E+02	6.9983E+02	6.2312E+02	6.1053E+02	6.0641E+02
	Std	2.1198E+01	1.8174E+01	2.9973E+01	2.7373E+01	2.5620E+01	4.0504E+01	3.5778E+01	2.9089E+01	3.2535E+01
F5	Mean	6.1005E+02	6.9296E+02	6.3247E+02	6.1092E+02	6.2796E+02	6.5013E+02	6.0354E+02	6.1386E+02	6.0985E+02
	Std	3.7657E+00	4.2741E+00	1.5057E+01	6.3865E+00	1.1342E+01	9.7149E+00	1.8171E+00	5.2131E+00	3.8177E+00
F6	Mean	9.3385E+02	1.3958E+03	9.8626E+02	9.2032E+02	9.6765E+02	1.0571E+03	9.3079E+02	8.8468E+02	9.1093E+02
	Std	3.2566E+01	4.4109E+01	4.7633E+01	5.8043E+01	2.1432E+01	9.1748E+01	3.6571E+01	3.8042E+01	4.7911E+01
F7	Mean	9.1539E+02	1.1443E+03	9.5242E+02	9.0265E+02	9.7649E+02	9.4529E+02	9.1396E+02	9.2001E+02	9.0025E+02
	Std	1.7426E+01	1.8531E+01	2.3103E+01	2.6319E+01	2.0179E+01	2.5802E+01	3.9981E+01	2.3627E+01	2.4298E+01
F8	Mean	1.8328E+03	1.1332E+04	5.2740E+03	2.5801E+03	4.3930E+03	4.8964E+03	1.5029E+03	3.8044E+03	2.6754E+03
	Std	5.1037E+02	8.5040E+02	1.2500E+03	6.7613E+02	2.3030E+03	1.0294E+03	5.3067E+02	1.5689E+03	1.0880E+03
F9	Mean	6.5327E+03	8.7734E+03	5.4231E+03	4.0967E+03	5.9987E+03	4.9235E+03	8.1256E+03	5.0907E+03	7.7680E+03
	Std	3.1648E+02	3.8044E+02	5.5703E+02	7.0095E+02	6.1878E+02	7.3792E+02	8.7850E+02	8.3030E+02	6.8689E+02
F10	Mean	1.2251E+03	8.1354E+03	1.4556E+03	1.2666E+03	1.2969E+03	1.2424E+03	1.5461E+03	1.3486E+03	1.2274E+03
	Std	4.1769E+01	1.2557E+03	1.7634E+02	6.4411E+01	2.4359E+01	4.1102E+01	1.3095E+02	5.2356E+01	4.7851E+01
F11	Mean	2.2907E+05	1.1553E+10	7.4455E+06	1.8087E+06	1.0270E+07	1.4430E+07	3.1656E+06	3.4937E+06	6.3557E+05
	Std	4.8448E+05	2.3468E+09	3.9168E+06	1.2467E+06	4.5826E+06	9.1147E+06	1.7121E+06	2.3837E+06	6.9445E+05
F12	Mean	4.4152E+03	6.3210E+09	9.9207E+04	1.8668E+04	8.4971E+04	3.5706E+05	5.3740E+04	3.0331E+04	1.2757E+04
	Std	1.1481E+03	2.0084E+09	9.7203E+04	1.6949E+04	3.7025E+04	2.6284E+05	4.3209E+04	2.4899E+04	9.8466E+03
F13	Mean	1.4912E+03	3.5607E+06	1.7796E+05	1.9064E+04	1.5782E+03	1.1903E+05	7.7716E+04	1.1756E+05	1.5478E+03
	Std	1.6018E+01	2.1416E+06	1.5004E+05	3.0373E+04	2.2389E+01	9.5763E+04	7.8589E+04	6.6258E+04	3.7729E+01
F14	Mean	1.7404E+03	3.8304E+08	6.7216E+03	6.7757E+03	3.1003E+03	6.4870E+04	2.3249E+04	1.7685E+04	2.1783E+03
	Std	7.3243E+01	1.7603E+08	9.3464E+03	5.3179E+03	6.9898E+02	4.6737E+04	1.5695E+04	1.4606E+04	4.4330E+02
F15	Mean	2.6400E+03	5.7183E+03	2.9120E+03	2.5805E+03	2.7935E+03	2.8869E+03	3.0223E+03	2.5149E+03	2.6980E+03
	Std	1.9470E+02	4.2066E+02	2.1913E+02	2.3445E+02	2.6042E+02	3.4557E+02	4.5739E+02	3.6956E+02	3.9899E+02
F16	Mean	1.9960E+03	4.1221E+03	2.0609E+03	2.1430E+03	1.9529E+03	2.4306E+03	2.0994E+03	2.2498E+03	2.0069E+03
	Std	9.9731E+01	6.9358E+02	1.6135E+02	1.9867E+02	8.6418E+01	2.4508E+02	2.1612E+02	2.3116E+02	1.6569E+02
F17	Mean	2.1619E+03	4.8999E+07	9.1343E+05	3.7393E+05	5.3031E+04	1.7202E+06	1.6612E+06	1.0963E+06	5.3431E+04
	Std	1.4662E+02	2.2131E+07	6.3727E+05	4.2955E+05	3.1923E+04	1.4746E+06	1.3883E+06	1.3089E+06	6.3402E+04
F18	Mean	1.9810E+03	4.6237E+08	1.0840E+04	7.9839E+03	3.6838E+03	1.2639E+05	1.9488E+04	2.0988E+04	2.2645E+03
	Std	3.2536E+01	1.9935E+08	1.6080E+04	6.2622E+03	1.1376E+03	1.2746E+05	1.9183E+04	1.8005E+04	4.5466E+02
F19	Mean	2.4108E+03	3.0503E+03	2.4315E+03	2.4228E+03	2.3153E+03	2.6949E+03	2.4655E+03	2.4782E+03	2.3647E+03
	Std	9.6138E+01	1.1700E+02	1.1524E+02	1.5271E+02	1.2659E+02	2.3423E+02	2.2869E+02	1.9515E+02	1.2554E+02
F20	Mean	2.4186E+03	2.7441E+03	2.4599E+03	2.3937E+03	2.4825E+03	2.4762E+03	2.4255E+03	2.4054E+03	2.4033E+03
	Std	2.2343E+01	3.0286E+01	5.0465E+01	2.3800E+01	1.7485E+01	3.5582E+01	3.4680E+01	2.6647E+01	3.5017E+01
F21	Mean	2.3151E+03	8.7511E+03	2.3830E+03	2.3225E+03	2.3497E+03	5.9919E+03	5.6818E+03	5.9713E+03	2.9507E+03
	Std	4.4989E+00	4.5302E+02	2.6705E+01	4.0520E+00	8.6606E+00	1.7849E+03	3.6644E+03	1.3156E+03	1.9093E+03
F22	Mean	2.7789E+03	3.3347E+03	2.8379E+03	2.7838E+03	2.8809E+03	2.9811E+03	2.7870E+03	2.7469E+03	2.7543E+03
	Std	2.1628E+01	5.7297E+01	2.8556E+01	4.2029E+01	2.5685E+01	7.6333E+01	3.4573E+01	2.8310E+01	3.2952E+01
F23	Mean	2.9332E+03	3.6139E+03	3.0239E+03	2.9535E+03	3.0373E+03	3.1853E+03	3.0217E+03	2.9192E+03	2.9242E+03
	Std	2.0394E+01	6.8666E+01	2.5484E+01	3.3418E+01	2.8476E+01	7.2735E+01	3.3198E+01	2.7065E+01	3.6642E+01
F24	Mean	2.8920E+03	4.5320E+03	3.0107E+03	2.9261E+03	2.9194E+03	2.9017E+03	2.9204E+03	2.9165E+03	2.9193E+03
	Std	7.3073E+00	1.9992E+02	2.6710E+01	2.3311E+01	1.4611E+01	1.8749E+01	1.9888E+01	1.9527E+01	2.8156E+01
F25	Mean	4.9497E+03	1.0766E+04	4.8908E+03	4.4687E+03	4.5373E+03	5.8179E+03	4.9403E+03	4.9278E+03	4.8463E+03
	Std	2.0883E+02	6.9747E+02	1.2853E+03	1.0834E+03	1.3469E+03	1.5055E+03	3.6175E+02	2.9453E+02	4.9652E+02
F26	Mean	3.2163E+03	4.0402E+03	3.2019E+03	3.2553E+03	3.2935E+03	3.2000E+03	3.2260E+03	3.2285E+03	3.2334E+03
	Std	1.3634E+01	1.4942E+02	1.0142E+01	1.8488E+01	2.3378E+01	2.7369E-04	1.0176E+01	1.4798E+01	2.3500E+01
F27	Mean	3.2327E+03	6.5104E+03	3.3640E+03	3.2855E+03	3.2872E+03	3.2983E+03	3.3209E+03	3.2966E+03	3.2658E+03
	Std	1.8858E+01	2.8257E+02	5.9363E+01	2.9751E+01	2.8043E+01	3.9827E+00	4.2667E+01	5.5261E+01	3.4792E+01
F28	Mean	3.8706E+03	7.3612E+03	3.9872E+03	3.8560E+03	4.0502E+03	3.8922E+03	3.8258E+03	3.9143E+03	3.7879E+03
	Std	1.3397E+02	7.1598E+02	1.8252E+02	1.9965E+02	1.9511E+02	2.7834E+02	1.9617E+02	2.1309E+02	1.7999E+02
F29	Mean	1.6128E+04	1.2045E+09	1.3584E+05	2.7234E+04	4.1441E+05	9.3769E+04	1.2126E+05	2.8545E+04	1.9738E+04
	Std	5.9102E+03	5.3722E+08	9.8972E+04	1.4355E+04	1.9945E+05	7.6330E+04	1.3887E+05	2.5723E+04	8.9179E+03

**Table A3:** Statistical measures of AMBWO with other algorithms using CEC2017, D = 50.

Function No.	Index	AMBWO	BWO	HBWO-JS	FDBARO	MCOA	DETDO	BEESO	DTSMA	IDE-EDA
F1	Mean	1.6177E+08	1.0760E+11	2.9517E+09	1.6816E+09	1.5388E+09	2.6261E+07	4.7900E+08	1.3164E+09	8.3553E+08
	Std	7.4677E+07	4.8606E+09	8.1012E+08	9.6952E+08	5.2671E+08	1.2635E+07	2.3685E+08	1.6102E+09	5.0491E+08
F2	Mean	5.7588E+04	2.2827E+05	1.9016E+05	1.4594E+05	1.8495E+05	1.6247E+05	1.9110E+05	1.9676E+05	1.0453E+05
	Std	1.3755E+04	2.7743E+04	2.4160E+04	1.5557E+04	2.2025E+04	5.9107E+04	2.0729E+04	5.0661E+04	2.0000E+04
F3	Mean	6.2367E+02	3.4748E+04	1.2663E+03	8.6348E+02	9.9537E+02	6.2512E+02	8.0700E+02	6.6342E+02	7.7330E+02
	Std	5.4058E+01	3.1006E+03	2.3599E+02	1.5979E+02	1.2145E+02	5.5387E+01	8.8976E+01	7.4195E+01	9.5921E+01
F4	Mean	8.4093E+02	1.1988E+03	8.7151E+02	7.7372E+02	9.5992E+02	8.3067E+02	8.4064E+02	7.9253E+02	7.8071E+02
	Std	3.8107E+01	1.7290E+01	3.5286E+01	3.3800E+01	2.8508E+01	3.3509E+01	5.8250E+01	5.9389E+01	5.2267E+01
F5	Mean	6.3008E+02	7.0291E+02	6.5517E+02	6.2763E+02	6.6552E+02	6.6008E+02	6.1371E+02	6.3308E+02	6.3136E+02
	Std	6.4688E+00	2.4266E+00	1.1448E+01	7.5583E+00	1.4839E+01	6.1693E+00	4.2562E+00	9.1477E+00	9.5219E+00
F6	Mean	1.3082E+03	1.9951E+03	1.3930E+03	1.2604E+03	1.2614E+03	1.4215E+03	1.1643E+03	1.1686E+03	1.2692E+03
	Std	5.8485E+01	4.5939E+01	1.2583E+02	1.0279E+02	4.2440E+01	1.1250E+02	6.0647E+01	6.2580E+01	9.2439E+01
F7	Mean	1.1403E+03	1.5060E+03	1.1863E+03	1.0675E+03	1.2562E+03	1.1438E+03	1.1129E+03	1.0835E+03	1.0934E+03
	Std	3.3575E+01	2.9210E+01	2.5855E+01	3.5197E+01	4.2764E+01	4.0126E+01	5.2071E+01	4.7052E+01	6.9187E+01
F8	Mean	1.0599E+04	3.8617E+04	1.9361E+04	9.5912E+03	2.0699E+04	1.4922E+04	5.6864E+03	1.5392E+04	1.3563E+04
	Std	2.9619E+03	2.4705E+03	2.4153E+03	1.8695E+03	7.1856E+03	2.1471E+03	2.0764E+03	6.7622E+03	5.7953E+03
F9	Mean	1.2555E+04	1.5115E+04	9.4833E+03	7.3283E+03	1.0513E+04	8.7680E+03	1.4861E+04	9.5096E+03	1.4083E+04
	Std	3.9158E+02	4.8307E+02	8.9218E+02	9.2528E+02	9.3410E+02	9.6809E+02	6.1048E+02	1.3620E+03	8.7858E+02
F10	Mean	1.4997E+03	2.2519E+04	3.3529E+03	1.8196E+03	1.8408E+03	1.5020E+03	5.5297E+03	1.8572E+03	1.6359E+03
	Std	6.8123E+01	2.1468E+03	1.0078E+03	2.4716E+02	2.4231E+02	9.0952E+01	2.2712E+03	5.8645E+02	2.0093E+02
F11	Mean	1.1695E+07	6.6373E+10	3.7295E+08	5.5198E+07	2.0650E+08	1.1191E+08	6.7770E+07	4.2407E+07	1.9331E+07
	Std	5.2150E+06	1.0474E+10	1.9680E+08	3.4255E+07	5.6261E+07	6.6869E+07	2.3871E+07	3.3308E+07	1.3734E+07
F12	Mean	7.3722E+04	3.4316E+10	2.6454E+07	4.6328E+04	6.4933E+06	9.1836E+05	4.1782E+05	4.6595E+04	1.9159E+04
	Std	4.3094E+04	7.8416E+09	1.6199E+07	2.1829E+04	5.3351E+06	5.1227E+05	3.7001E+05	1.9569E+04	8.5970E+03
F13	Mean	1.7146E+03	5.7697E+07	1.4870E+06	2.0445E+05	3.0832E+04	1.2209E+06	7.2006E+05	4.8895E+05	3.1716E+04
	Std	6.1457E+01	2.8216E+07	1.2974E+06	1.6441E+05	4.5138E+04	7.2064E+05	5.4096E+05	3.4568E+05	5.0880E+04
F14	Mean	5.9219E+03	5.6450E+09	2.8240E+05	1.1854E+04	1.5710E+05	2.3579E+05	4.8408E+04	2.1630E+04	9.5454E+03
	Std	4.6169E+03	1.5262E+09	3.8682E+05	6.5447E+03	7.0100E+04	1.3584E+05	2.3248E+04	1.0693E+04	5.0306E+03
F15	Mean	3.6117E+03	9.0639E+03	3.7700E+03	3.2711E+03	4.0011E+03	3.8705E+03	4.7001E+03	3.5595E+03	3.4157E+03
	Std	2.9495E+02	6.3153E+02	5.3721E+02	4.6400E+02	3.7846E+02	6.2476E+02	6.4720E+02	4.3529E+02	6.3546E+02
F16	Mean	3.1473E+03	7.6406E+03	3.1467E+03	3.0490E+03	3.1176E+03	3.4021E+03	3.8158E+03	3.2405E+03	3.0439E+03
	Std	1.9096E+02	1.7281E+03	3.5286E+02	3.6160E+02	2.8572E+02	3.3467E+02	4.5774E+02	2.6238E+02	4.1517E+02
F17	Mean	3.8616E+04	1.3309E+08	5.4849E+06	1.9573E+06	9.2870E+05	2.6297E+06	8.1545E+06	5.3958E+06	3.3450E+05
	Std	3.0426E+04	5.5830E+07	3.4092E+06	1.3600E+06	9.1938E+05	1.4542E+06	5.6879E+06	3.8273E+06	3.2748E+05
F18	Mean	5.4080E+03	3.1818E+09	4.1769E+04	1.7136E+04	1.4939E+05	4.1891E+05	4.2105E+04	2.1521E+04	1.4402E+04
	Std	5.4730E+03	9.5366E+08	2.3176E+04	9.1551E+03	6.9901E+04	3.0523E+05	2.7273E+04	1.7566E+04	1.0452E+04
F19	Mean	3.3105E+03	4.1511E+03	3.1351E+03	3.0863E+03	2.9179E+03	3.4296E+03	4.0253E+03	3.2624E+03	3.5070E+03
	Std	1.9807E+02	2.1340E+02	2.3355E+02	3.0402E+02	2.4607E+02	3.6759E+02	2.5594E+02	3.2848E+02	4.1377E+02
F20	Mean	2.6299E+03	3.2013E+03	2.7017E+03	2.5403E+03	2.7159E+03	2.6917E+03	2.6438E+03	2.5514E+03	2.5558E+03
	Std	2.7338E+01	4.9204E+01	4.7554E+01	3.7724E+01	2.8666E+01	6.5409E+01	6.0701E+01	5.1553E+01	5.0595E+01
F21	Mean	1.3199E+04	1.6811E+04	1.1758E+04	9.3144E+03	1.2016E+04	1.0311E+04	1.6462E+04	1.1165E+04	1.3912E+04
	Std	2.8422E+03	4.6443E+02	1.5998E+03	8.0402E+02	3.0329E+03	1.1822E+03	7.6533E+02	1.1438E+03	4.1851E+03
F22	Mean	3.0791E+03	4.1201E+03	3.2112E+03	3.0494E+03	3.2852E+03	3.2470E+03	3.0966E+03	2.9921E+03	3.0709E+03
	Std	4.2448E+01	6.3472E+01	3.9096E+01	8.3388E+01	5.5070E+01	1.8318E+02	5.7080E+01	4.5665E+01	7.9006E+01
F23	Mean	3.2268E+03	4.5592E+03	3.4728E+03	3.2475E+03	3.4313E+03	3.6425E+03	3.3168E+03	3.0900E+03	3.1850E+03
	Std	4.2632E+01	1.2392E+02	6.8758E+01	6.3737E+01	4.6522E+01	1.1193E+02	5.8209E+01	4.7077E+01	6.4620E+01
F24	Mean	3.1306E+03	1.4112E+04	3.7631E+03	3.3951E+03	3.3555E+03	3.0887E+03	3.3228E+03	3.1986E+03	3.2351E+03
	Std	2.8178E+01	8.5999E+02	2.1692E+02	1.2734E+02	7.2920E+01	3.0208E+01	1.1656E+02	8.6187E+01	6.3615E+01
F25	Mean	7.3345E+03	1.6771E+04	8.7742E+03	7.2200E+03	5.3423E+03	8.4077E+03	7.2269E+03	6.8288E+03	7.5698E+03
	Std	4.4692E+02	3.9821E+02	2.2651E+03	1.9993E+03	1.7419E+03	1.9886E+03	6.0464E+02	9.5545E+02	1.2071E+03
F26	Mean	3.4013E+03	6.1491E+03	3.2000E+03	3.7051E+03	3.9379E+03	3.2000E+03	3.5011E+03	3.4775E+03	3.5901E+03
	Std	7.0138E+01	3.7875E+02	8.6614E-05	1.1161E+02	1.2020E+02	2.8754E-04	8.8877E+01	8.9360E+01	1.3634E+02
F27	Mean	3.4299E+03	1.2498E+04	3.9664E+03	3.9885E+03	3.8706E+03	3.2994E+03	4.2404E+03	4.4824E+03	3.6008E+03
	Std	5.5086E+01	4.2700E+02	5.8126E+02	2.7237E+02	1.2990E+02	3.3483E+00	4.8816E+02	8.9043E+02	1.1441E+02
F28	Mean	4.7937E+03	3.3087E+04	4.9600E+03	4.5856E+03	5.4516E+03	5.0600E+03	4.6215E+03	4.9041E+03	4.6974E+03
	Std	2.7612E+02	2.1076E+04	4.1172E+02	3.8632E+02	3.0194E+02	6.9456E+02	3.7549E+02	3.5197E+02	2.8974E+02
F29	Mean	3.7310E+06	4.8982E+09	1.4888E+07	5.9970E+06	3.7123E+07	6.9682E+05	7.2956E+06	8.7295E+06	2.5546E+06
	Std	1.3221E+06	1.1280E+09	4.4237E+06	1.7791E+06	7.2652E+06	5.2275E+05	2.4016E+06	2.7972E+06	1.0421E+06

**Table A4:** Statistical measures of AMBWO with other algorithms using CEC2017, D = 100.

Function No.	Index	AMBWO	BWO	HBWO-JS	FDBARO	MCOA	DETDO	BEESO	DTSMA	IDE-EDA
F1	Mean	6.3327E+09	2.5926E+11	4.9244E+10	4.4785E+10	2.7936E+10	1.0467E+09	1.4836E+10	2.7712E+10	2.7645E+10
	Std	1.1356E+09	7.2466E+09	9.0983E+09	8.2328E+09	4.7376E+09	3.5405E+08	3.5703E+09	8.6767E+09	6.9256E+09
F2	Mean	2.5257E+05	3.7849E+05	3.5090E+05	3.5433E+05	3.4010E+05	4.3695E+05	4.4236E+05	7.0106E+05	3.0626E+05
	Std	1.7978E+04	4.4897E+04	1.9282E+04	3.3709E+04	1.6480E+04	1.9794E+05	6.7381E+04	1.3100E+05	3.9361E+04
F3	Mean	1.5880E+03	1.0172E+05	7.2331E+03	4.6713E+03	4.9113E+03	1.1687E+03	3.0840E+03	1.9418E+03	3.1085E+03
	Std	2.2730E+02	6.4109E+03	1.4923E+03	1.0160E+03	7.9313E+02	1.4057E+02	6.6112E+02	6.7023E+02	5.1438E+02
F4	Mean	1.5561E+03	2.1141E+03	1.5307E+03	1.3684E+03	1.7588E+03	1.3641E+03	1.4894E+03	1.3561E+03	1.3516E+03
	Std	5.2707E+01	3.2626E+01	5.0044E+01	7.1562E+01	5.9529E+01	5.8005E+01	7.7147E+01	9.3367E+01	1.0443E+02
F5	Mean	6.6588E+02	7.1235E+02	6.7735E+02	6.5194E+02	6.8787E+02	6.6772E+02	6.4138E+02	6.6545E+02	6.5435E+02
	Std	5.7624E+00	1.6383E+00	4.6773E+00	5.0996E+00	5.9330E+00	3.6585E+00	5.4966E+00	7.3358E+00	7.5030E+00
F6	Mean	2.6163E+03	3.9120E+03	2.8383E+03	2.7591E+03	2.2701E+03	2.9529E+03	2.0610E+03	2.4960E+03	2.7570E+03
	Std	1.6346E+02	5.4178E+01	2.4286E+02	2.4695E+02	8.7502E+01	1.3350E+02	1.2603E+02	2.4716E+02	1.6972E+02
F7	Mean	1.9110E+03	2.5934E+03	1.9847E+03	1.7533E+03	2.1223E+03	1.7769E+03	1.7794E+03	1.6244E+03	1.7334E+03
	Std	7.1723E+01	3.3347E+01	5.6610E+01	1.0261E+02	7.8386E+01	6.9099E+01	8.2214E+01	1.0076E+02	1.0491E+02
F8	Mean	5.1121E+04	8.0281E+04	4.7895E+04	3.2889E+04	6.3466E+04	3.2937E+04	3.4264E+04	4.4446E+04	5.5757E+04
	Std	7.9911E+03	3.4937E+03	3.1292E+03	3.5637E+03	6.8037E+03	3.1702E+03	6.3130E+03	9.0145E+03	1.0271E+04
F9	Mean	2.8997E+04	3.2258E+04	2.3415E+04	1.8451E+04	2.6070E+04	2.0080E+04	3.2374E+04	2.2292E+04	3.0853E+04
	Std	5.6950E+02	7.3069E+02	1.5705E+03	1.1254E+03	8.1972E+02	2.4561E+03	7.5655E+02	2.3584E+03	1.4009E+03
F10	Mean	1.9367E+04	3.4062E+05	1.4374E+05	6.9795E+04	1.0483E+05	4.3490E+04	1.6118E+05	6.6263E+04	3.2803E+04
	Std	6.3882E+03	6.4165E+04	2.5931E+04	1.7342E+04	1.7394E+04	1.2275E+04	2.9049E+04	2.3384E+04	1.0781E+04
F11	Mean	6.2009E+08	1.9353E+11	1.0288E+10	2.5792E+09	3.9879E+09	5.8193E+08	1.5037E+09	1.6170E+09	1.7304E+09
	Std	2.2162E+08	1.2375E+10	2.6214E+09	1.4128E+09	9.4985E+08	2.2422E+08	5.1882E+08	1.1105E+09	6.5737E+08
F12	Mean	1.1467E+06	4.3460E+10	4.4952E+08	1.0417E+07	6.6581E+07	1.8567E+06	5.9174E+06	8.6696E+06	1.4303E+06
	Std	3.9660E+05	3.7334E+09	1.5320E+08	6.4708E+06	2.5560E+07	1.0007E+06	3.9914E+06	2.6737E+07	1.6823E+06
F13	Mean	1.2152E+05	7.8303E+07	7.8633E+06	3.2679E+06	2.0741E+06	4.4920E+06	1.2018E+07	5.2424E+06	9.7535E+05
	Std	1.1935E+05	1.5824E+07	2.9861E+06	1.3877E+06	1.1120E+06	2.0658E+06	6.1598E+06	3.0808E+06	5.6479E+05
F14	Mean	7.1743E+04	2.2967E+10	3.3914E+07	1.2722E+05	5.9969E+06	5.9253E+05	6.5153E+05	3.6391E+05	2.4561E+04
	Std	2.3637E+04	2.1414E+09	1.7579E+07	9.8502E+04	3.5919E+06	4.4061E+05	5.2700E+05	9.3021E+05	1.0708E+04
F15	Mean	7.8300E+03	2.2494E+04	8.5095E+03	6.4568E+03	9.8405E+03	7.8922E+03	1.0534E+04	6.5250E+03	6.5317E+03
	Std	5.8755E+02	1.4697E+03	9.5668E+02	5.5044E+02	6.8658E+02	1.7390E+03	6.7568E+02	8.1905E+02	8.9249E+02
F16	Mean	5.7370E+03	5.9719E+06	6.8009E+03	5.1231E+03	6.9173E+03	7.0233E+03	7.9085E+03	5.5822E+03	5.4236E+03
	Std	3.6520E+02	2.8089E+06	9.8134E+02	6.5173E+02	3.5522E+02	1.2241E+03	6.1974E+02	5.7220E+02	7.5629E+02
F17	Mean	2.8315E+05	2.0096E+08	7.5536E+06	3.9970E+06	4.9590E+06	5.0398E+06	2.0504E+07	8.8411E+06	1.5446E+06
	Std	2.0068E+05	7.0034E+07	4.3819E+06	2.0264E+06	1.7880E+06	2.1489E+06	9.2447E+06	4.3493E+06	8.6524E+05
F18	Mean	3.8216E+05	2.2847E+10	2.8581E+07	2.7303E+05	9.6529E+06	2.5249E+06	2.1873E+06	2.3225E+05	1.2665E+05
	Std	2.4870E+05	2.2995E+09	1.4715E+07	1.6187E+05	5.8221E+06	1.4053E+06	1.3997E+06	3.5170E+05	1.1102E+05
F19	Mean	6.4336E+03	7.7368E+03	6.0375E+03	5.1266E+03	5.7250E+03	5.6644E+03	7.7105E+03	5.6861E+03	6.9669E+03
	Std	3.1321E+02	2.4946E+02	4.2161E+02	4.4334E+02	5.1512E+02	6.4658E+02	3.3415E+02	7.7533E+02	6.5034E+02
F20	Mean	3.3693E+03	4.7484E+03	3.5623E+03	3.1717E+03	3.5454E+03	3.6061E+03	3.3349E+03	3.1158E+03	3.2377E+03
	Std	6.8968E+01	1.1330E+02	6.2267E+01	8.3532E+01	6.1305E+01	1.8625E+02	9.5172E+01	1.0769E+02	1.0807E+02
F21	Mean	3.0973E+04	3.4747E+04	2.5624E+04	2.1151E+04	2.9268E+04	2.3037E+04	3.4230E+04	2.4199E+04	3.3349E+04
	Std	9.2048E+02	7.6641E+02	1.6519E+03	1.6634E+03	7.6761E+02	2.2410E+03	1.2764E+03	2.3606E+03	1.3509E+03
F22	Mean	3.8392E+03	6.1139E+03	4.0702E+03	3.7710E+03	4.3857E+03	4.3432E+03	3.7391E+03	3.5016E+03	3.8067E+03
	Std	5.3012E+01	1.8866E+02	1.3416E+02	1.1043E+02	1.1924E+02	2.2078E+02	1.1123E+02	1.0747E+02	1.2681E+02
F23	Mean	4.3569E+03	9.4826E+03	5.0811E+03	4.7017E+03	5.3333E+03	5.7881E+03	4.3518E+03	4.0519E+03	4.5790E+03
	Std	8.7103E+01	3.6218E+02	5.2632E+02	1.6129E+02	1.4098E+02	3.6789E+02	1.2108E+02	1.2156E+02	2.0146E+02
F24	Mean	4.3911E+03	2.7535E+04	7.1272E+03	5.9784E+03	5.8641E+03	3.6838E+03	5.6917E+03	5.6049E+03	5.4381E+03
	Std	1.9849E+02	8.8758E+02	6.6305E+02	5.4514E+02	3.6980E+02	8.5012E+01	5.2894E+02	8.9054E+02	4.4048E+02
F25	Mean	1.7327E+04	5.1414E+04	2.6261E+04	2.2808E+04	2.2219E+04	2.6701E+04	1.7080E+04	1.3560E+04	2.1236E+04
	Std	1.1449E+03	1.0817E+03	1.8265E+03	2.6503E+03	3.7300E+03	5.3811E+03	1.0454E+03	1.0609E+03	2.3239E+03
F26	Mean	3.7074E+03	1.2295E+04	3.3341E+03	4.3778E+03	4.9654E+03	3.2000E+03	3.8511E+03	3.6845E+03	4.0579E+03
	Std	7.5296E+01	1.0021E+03	4.2263E+02	2.2342E+02	1.8447E+02	3.8615E-04	9.0906E+01	8.2485E+01	1.5842E+02
F27	Mean	4.7174E+03	2.7478E+04	7.1334E+03	8.5727E+03	7.4906E+03	3.3000E+03	9.4512E+03	1.0350E+04	7.2199E+03
	Std	3.7315E+02	8.6948E+02	2.8265E+03	9.3891E+02	7.6148E+02	4.9078E-04	1.4735E+03	3.9258E+03	9.5199E+02
F28	Mean	9.2436E+03	4.8654E+05	9.5049E+03	8.3109E+03	1.0773E+04	7.6051E+03	8.9554E+03	8.2648E+03	8.4408E+03
	Std	5.7266E+02	1.8296E+05	8.2922E+02	4.7535E+02	5.2875E+02	1.2104E+03	8.1304E+02	6.3833E+02	6.6451E+02
F29	Mean	7.5322E+06	4.1109E+10	4.5189E+08	2.1622E+07	1.0137E+08	9.8133E+06	2.1242E+07	1.6374E+07	5.8524E+06
	Std	2.9995E+06	3.5337E+09	2.2039E+08	1.0640E+07	3.0759E+07	5.3139E+06	1.2474E+07	9.1441E+06	3.6718E+06

**Table A5:** Statistical measures of AMBWO with other algorithms using CEC2022, D = 10.

Function No.	Index	AMBWO	BWO	HBWO-JS	FDBARO	MCOA	DETDO	BEESO	DTSMA	IDE-EDA
F1	Mean	3.0000E+02	8.2345E+03	1.9302E+03	3.1321E+02	3.0111E+02	3.0016E+02	1.8067E+03	3.0066E+02	3.0000E+02
	Std	2.8964E-08	2.4800E+03	7.1165E+02	2.2166E+01	1.3370E+00	3.2187E-01	7.9442E+02	1.5675E+00	5.1148E-05
F2	Mean	4.0477E+02	8.3004E+02	4.0463E+02	4.0519E+02	4.0121E+02	4.1982E+02	4.0551E+02	4.0775E+02	4.0647E+02
	Std	2.8729E+00	1.4031E+02	8.7906E+00	1.2342E+01	1.9688E+00	3.1292E+01	2.7405E+00	1.9668E+00	1.2537E+01
F3	Mean	6.0001E+02	6.4763E+02	6.0007E+02	6.0003E+02	6.0054E+02	6.1310E+02	6.0004E+02	6.0022E+02	6.0001E+02
	Std	9.2085E-03	6.3169E+00	8.1402E-02	3.0351E-02	2.7111E-01	8.5960E+00	2.2902E-02	2.5970E-01	7.5071E-03
F4	Mean	8.0828E+02	8.4843E+02	8.1463E+02	8.1701E+02	8.2007E+02	8.2607E+02	8.2281E+02	8.2026E+02	8.1011E+02
	Std	2.3490E+00	8.4294E+00	5.6584E+00	8.8698E+00	6.1276E+00	9.1911E+00	9.9810E+00	8.3000E+00	5.0185E+00
F5	Mean	9.0002E+02	1.4917E+03	9.0245E+02	9.0806E+02	9.0006E+02	1.0795E+03	9.0010E+02	9.0261E+02	9.0049E+02
	Std	8.2948E-02	1.0561E+02	8.0498E+00	2.4549E+01	5.4274E-02	2.3145E+02	1.4856E-01	5.3963E+00	1.9866E+00
F6	Mean	1.8009E+03	2.5657E+06	2.3392E+03	2.0771E+03	1.8345E+03	2.3185E+04	4.7974E+03	4.8874E+03	1.8120E+03
	Std	7.4999E-01	1.7517E+06	5.7908E+02	9.2144E+02	2.1696E+01	8.6179E+04	2.2146E+03	2.0271E+03	1.4851E+01
F7	Mean	2.0174E+03	2.1032E+03	2.0220E+03	2.0121E+03	2.0170E+03	2.0394E+03	2.0179E+03	2.0204E+03	2.0174E+03
	Std	6.1575E+00	1.7682E+01	7.6467E+00	1.1079E+01	7.1006E+00	2.1840E+01	8.9349E+00	5.2069E+00	9.5456E+00
F8	Mean	2.2156E+03	2.2438E+03	2.2209E+03	2.2192E+03	2.2176E+03	2.2248E+03	2.2235E+03	2.2211E+03	2.2181E+03
	Std	5.8494E+00	5.8792E+00	4.5694E+00	3.8065E+00	6.5922E+00	3.7003E+00	4.8648E+00	4.8393E+00	9.7142E+00
F9	Mean	2.5293E+03	2.7036E+03	2.5312E+03	2.5293E+03	2.5293E+03	2.4997E+03	2.5293E+03	2.5293E+03	2.5293E+03
	Std	5.9285E-09	2.1851E+01	7.3821E+00	2.7215E-03	2.3254E-02	5.1468E+01	8.0652E-07	3.2031E-06	1.2723E-12
F10	Mean	2.5111E+03	2.5659E+03	2.5152E+03	2.5120E+03	2.5379E+03	2.5769E+03	2.5045E+03	2.5047E+03	2.5302E+03
	Std	3.2798E+01	6.1119E+01	3.8153E+01	3.5179E+01	5.3824E+01	1.1207E+02	4.7705E+01	2.3283E+01	5.0352E+01
F11	Mean	2.6100E+03	3.3170E+03	2.6567E+03	2.6551E+03	2.6010E+03	2.8160E+03	2.8471E+03	2.6853E+03	2.9014E+03
	Std	3.8169E+01	2.2062E+02	1.0849E+02	1.0777E+02	3.8523E-01	1.3525E+02	1.0452E+02	1.2286E+02	4.1723E+00
F12	Mean	2.8617E+03	2.9079E+03	2.8634E+03	2.8656E+03	2.8642E+03	2.8949E+03	2.8641E+03	2.8634E+03	2.8646E+03
	Std	1.6732E+00	1.9255E+01	1.8405E+00	1.5455E+00	9.6806E-01	1.5698E+01	1.5934E+00	1.7575E+00	2.0918E+00

**Table A6:** Statistical measures of AMBWO with other algorithms using CEC2022, D = 20.

Function No.	Index	AMBWO	BWO	HBWO-JS	FDBARO	MCOA	DETDO	BEESO	DTSMA	IDE-EDA
F1	Mean	4.0907E+02	7.0632E+04	2.8140E+04	9.8391E+03	1.3059E+04	3.5938E+03	2.4220E+04	3.0638E+03	2.5299E+03
	Std	1.7718E+02	1.8536E+04	6.9360E+03	4.7757E+03	4.9409E+03	2.5189E+03	6.8823E+03	2.2493E+03	1.7396E+03
F2	Mean	4.4602E+02	2.2467E+03	5.0352E+02	4.6463E+02	4.5756E+02	4.4140E+02	4.5381E+02	4.5925E+02	4.5990E+02
	Std	1.2096E+01	2.5482E+02	4.2123E+01	3.3154E+01	1.3794E+01	2.7964E+01	1.6484E+01	2.4050E+01	1.4090E+01
F3	Mean	6.0362E+02	6.8116E+02	6.0844E+02	6.0277E+02	6.0931E+02	6.4148E+02	6.0065E+02	6.0431E+02	6.0185E+02
	Std	2.1475E+00	6.3361E+00	1.0923E+01	2.2075E+00	3.7856E+00	9.0678E+00	3.2717E-01	3.9264E+00	1.4637E+00
F4	Mean	8.4627E+02	9.7480E+02	8.7369E+02	8.5863E+02	8.7735E+02	8.7649E+02	9.0766E+02	8.6086E+02	8.4294E+02
	Std	1.0797E+01	9.8259E+00	1.2983E+01	1.6713E+01	1.2462E+01	1.6419E+01	1.7087E+01	2.0391E+01	1.5814E+01
F5	Mean	1.0586E+03	3.6688E+03	2.2967E+03	1.2891E+03	1.5493E+03	2.0356E+03	9.6588E+02	1.4996E+03	9.9463E+02
	Std	1.3500E+02	3.3149E+02	4.5557E+02	3.2922E+02	7.3568E+02	2.8448E+02	8.1460E+01	5.2069E+02	1.2544E+02
F6	Mean	1.8983E+03	1.3510E+09	6.2753E+03	5.8359E+03	3.8298E+04	8.0378E+03	2.5159E+04	1.5327E+04	2.8913E+03
	Std	3.8364E+01	4.1459E+08	4.6883E+03	4.4316E+03	2.0394E+04	8.1090E+03	2.2453E+04	8.2851E+03	1.8068E+03
F7	Mean	2.0618E+03	2.2258E+03	2.0935E+03	2.0654E+03	2.0586E+03	2.1372E+03	2.0594E+03	2.0611E+03	2.0538E+03
	Std	1.2769E+01	2.7864E+01	1.8739E+01	3.8658E+01	1.2172E+01	5.3982E+01	1.7507E+01	3.2929E+01	1.8091E+01
F8	Mean	2.2312E+03	2.2912E+03	2.2292E+03	2.2360E+03	2.2305E+03	2.2895E+03	2.2384E+03	2.2330E+03	2.2381E+03
	Std	2.4371E+00	3.3269E+01	1.5032E+00	3.5739E+01	1.6661E+00	6.2135E+01	7.6860E+00	9.5043E+00	3.1479E+01
F9	Mean	2.4808E+03	3.0736E+03	2.4919E+03	2.4832E+03	2.4843E+03	2.4763E+03	2.4809E+03	2.4811E+03	2.4808E+03
	Std	1.6572E-03	1.2985E+02	6.4394E+00	1.9646E+00	9.6462E-01	4.2778E+01	9.4089E-02	2.8362E-01	9.5116E-03
F10	Mean	2.6919E+03	3.8578E+03	2.6199E+03	2.5429E+03	2.6885E+03	3.6863E+03	2.8209E+03	2.9975E+03	2.8643E+03
	Std	4.7647E+02	1.1255E+03	2.3667E+02	1.0396E+02	4.2647E+02	6.4397E+02	2.6158E+02	7.3053E+02	9.0434E+02
F11	Mean	2.9061E+03	8.1868E+03	3.1310E+03	3.0580E+03	3.0685E+03	2.9137E+03	3.0398E+03	2.9532E+03	2.9175E+03
	Std	6.3646E+01	5.6602E+02	1.2630E+02	4.6555E+02	1.7719E+02	2.3790E+01	4.0621E+01	1.4085E+02	2.1295E+01
F12	Mean	2.9436E+03	3.3375E+03	2.9000E+03	2.9733E+03	2.9797E+03	2.9000E+03	2.9589E+03	2.9467E+03	2.9606E+03
	Std	8.7301E+00	9.5945E+01	6.5282E-05	1.9194E+01	1.0990E+01	2.2096E-04	1.8517E+01	6.7168E+00	1.9451E+01
